# Microglia regulate sleep through calcium-dependent modulation of norepinephrine transmission

**DOI:** 10.1038/s41593-023-01548-5

**Published:** 2024-01-18

**Authors:** Chenyan Ma, Bing Li, Daniel Silverman, Xinlu Ding, Anan Li, Chi Xiao, Ganghua Huang, Kurtresha Worden, Sandra Muroy, Wei Chen, Zhengchao Xu, Chak Foon Tso, Yixuan Huang, Yufan Zhang, Qingming Luo, Kaoru Saijo, Yang Dan

**Affiliations:** 1 Division of Neurobiology, Department of Molecular and Cell Biology, Helen Wills Neuroscience Institute, Howard Hughes Medical Institute, University of California, Berkeley, Berkeley, CA USA; 2 Biomedical Photonics, Wuhan National Laboratory for Optoelectronics, Huazhong University of Science and Technology, Wuhan, China; 3 Research Unit of Multimodal Cross Scale Neural Signal Detection and Imaging, Chinese Academy of Medical Sciences, HUST-Suzhou Institute for Brainmatics, JITRI, Suzhou, China; 4 Key Laboratory of Biomedical Engineering of Hainan Province, School of Biomedical Engineering, Hainan University, Haikou, China; 5 Division of Immunology and Pathogenesis, Department of Molecular and Cell Biology, Helen Wills Neuroscience Institute, University of California, Berkeley, Berkeley, CA USA; 6 Department of Physics, University of California, Berkeley, Berkeley, CA USA; 7 Sunnyvale, CA USA

**Keywords:** Sleep, Microglia

## Abstract

Sleep interacts reciprocally with immune system activity, but its specific relationship with microglia—the resident immune cells in the brain—remains poorly understood. Here, we show in mice that microglia can regulate sleep through a mechanism involving G_i_-coupled GPCRs, intracellular Ca^2+^ signaling and suppression of norepinephrine transmission. Chemogenetic activation of microglia G_i_ signaling strongly promoted sleep, whereas pharmacological blockade of G_i_-coupled P2Y12 receptors decreased sleep. Two-photon imaging in the cortex showed that P2Y12–G_i_ activation elevated microglia intracellular Ca^2+^, and blockade of this Ca^2+^ elevation largely abolished the G_i_-induced sleep increase. Microglia Ca^2+^ level also increased at natural wake-to-sleep transitions, caused partly by reduced norepinephrine levels. Furthermore, imaging of norepinephrine with its biosensor in the cortex showed that microglia P2Y12–G_i_ activation significantly reduced norepinephrine levels, partly by increasing the adenosine concentration. These findings indicate that microglia can regulate sleep through reciprocal interactions with norepinephrine transmission.

## Abstract (web summary)

Immune activity can influence sleep, but the role of microglia has remained unclear. Ma, Li and colleagues show that microglia can promote sleep through P2Y12–G_i_-coupled GPCR signaling, intracellular calcium increase and suppression of norepinephrine transmission.

## Main

Sleep has a vital role in brain health and function by facilitating multiple physiological processes, including homeostatic regulation of neuronal activity, synaptic strengths and clearance of metabolic waste products^1–4^. Microglia, the primary immune cells in the brain, have a key part in brain homeostasis by modulating neuronal activity, pruning synapses, and clearing cellular debris and harmful aggregates^5–11^. Both sleep disturbances and microglia dysfunction have been implicated in multiple neurodegenerative diseases^5,7,12–14^. However, the role of microglia in sleep regulation is only beginning to be investigated^15–17^. In addition to specific neuronal circuits controlling sleep^18^, several metabolic substances (for example, ATP and adenosine) and immune-modulating cytokines (for example, interleukin (IL)-1β and tumor necrosis factor α (TNFα)) have been shown to promote sleep^19^. Microglia are well suited for mediating such sleep-regulating effects, as they constantly survey the brain parenchyma with their motile processes, both sensing and responding to purinergic molecules and cytokines^5,8,9^.

In a healthy brain, microglia exist in a homeostatic state, characterized by ramified morphology and expression of specific genes supporting homeostatic functions^6,9^. One of these homeostatic genes encodes P2Y12, a G_i_-protein-coupled ATP/ADP receptor that is highly expressed specifically in microglia within the central nervous system^20^. P2Y12 is crucial for the function of microglia, particularly in their sensing and modulation of neuronal activity^20–23^, facilitation of experience-dependent plasticity^24^, and protection against epilepsy^21^ and ischemia-induced brain injury^23^. The ligands for P2Y12—ATP and ADP—as well as their metabolite, adenosine, are known to have important roles in homeostatic sleep regulation^25^.

We, thus, set out to study the role of microglia P2Y12–G_i_ signaling in regulating sleep. Using *Tmem119-CreERT2* mice for microglia-specific imaging and manipulation, we found that microglia P2Y12–G_i_ activation promoted sleep through a mechanism that depended on their intracellular Ca^2+^ signaling. Microglia Ca^2+^ activity was naturally higher during sleep than wakefulness, caused at least in part by a lower level of norepinephrine (NE). Conversely, microglia P2Y12–G_i_ activation reduced NE transmission, partly by increasing the level of extracellular adenosine.

## Results

### Activation of microglia G_i_ signaling promotes sleep

**Fig. 1:**
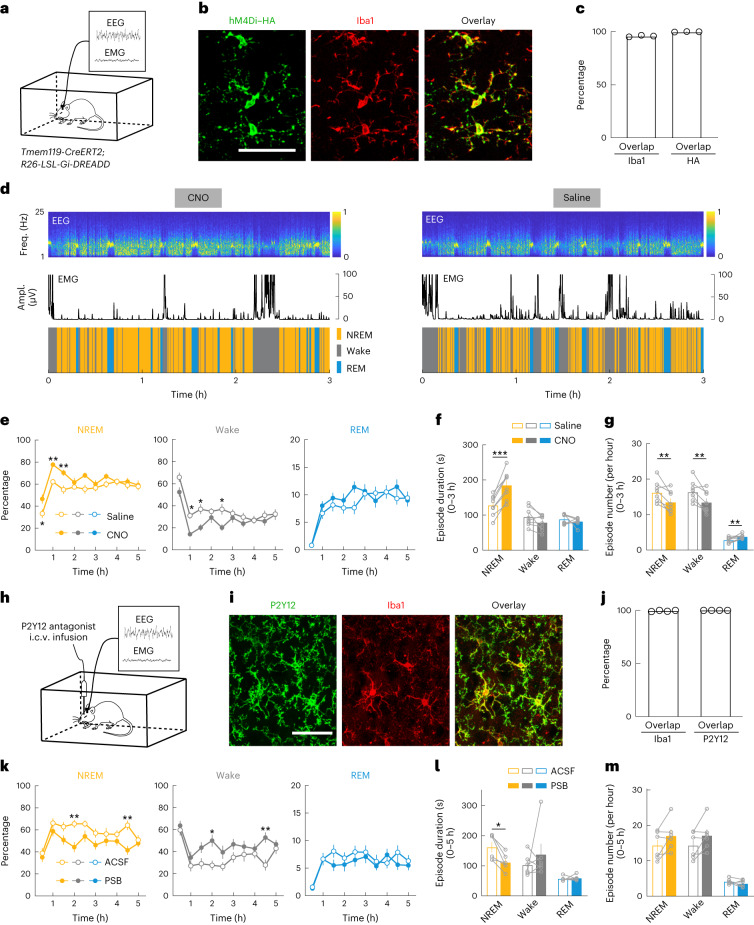
Microglia regulate sleep through P2Y12–G_i_ signaling. **a**, Schematic for the chemogenetic experiment. **b**, Confocal images from the prefrontal cortex showing hM4Di expression in Iba1^+^ microglia (Extended Data Fig. 1). Scale bar, 50 µm. **c**, Quantification of efficiency and specificity (mean ± s.e.m.; *n* = 3 mice). ‘Overlap’: HM4Di–HA^+^ and Iba1^+^. **d**, Example chemogenetic experiments. Shown are the EEG spectrogram (normalized by the maximum of each session; Freq., frequency), EMG amplitude (Ampl.) and brain states (color-coded). **e**, Summary of the percentages of time in each brain state following CNO and saline injection (mean ± s.e.m.; *n* = 8 mice: 3 female and 5 male, recorded between ZT6 and ZT11). *P < 0.05, **P < 0.01 (two-way ANOVA with Bonferroni correction; NREM: *P*_treatment_ < 0.0001, *P*_time_ < 0.0001, *P_0.5h_ = 0.044, **P_1h_ = 0.0087, **P_1.5h_ = 0.0097; wake: *P*_treatment_ < 0.0001, *P*_time_ < 0.0001, *P_1h_ = 0.020, *P_1.5h_ = 0.022, *P_2.5h_ = 0.024; REM: *P*_treatment_ = 0.13, *P*_time_ < 0.0001). **f**,**g**, Mean episode duration (**f**) and episode number per hour (**g**) for each brain state within 3 h of CNO or saline injection. Each circle indicates data from one mouse (mean ± s.e.m.; *n* = 8 mice). **P < 0.01, ***P < 0.001 (paired two-tailed *t*-test; in **f**, ***P = 0.0001; in **g**, **P_NREM_ = 0.0091, **P_wake_ = 0.0070, **P_REM_ = 0.0056). **h**, Schematic for P2Y12 antagonist infusion. **i**,**j**, Confocal images (**i**) and quantification (**j**) of P2Y12 expression in Iba1^+^ microglia in the prefrontal cortex (*n* = 4 mice). Scale bar, 50 μm. ‘Overlap’: Iba1^+^ and P2Y12^+^. **k**, Percentage of time in each brain state following PSB0739 (PSB) and ACSF infusion (mean ± s.e.m.; *n* = 6 mice: 1 female and 5 male). *P < 0.05, **P < 0.01 (two-way ANOVA with Bonferroni correction; NREM: *P*_treatment_ = 0.0005, *P*_time_ < 0.0001, **P_2h_ = 0.0053, **P_4.5h_ = 0.0021; wake: *P*_treatment_ = 0.0011, *P*_time_ < 0.0001, *P_2h_ = 0.014, **P_4.5h_ = 0.0037; REM: *P*_treatment_ = 0.23, *P*_time_ < 0.0001). **l**,**m**, Mean episode duration (**l**) and episode number per hour (**m**) for each brain state within 5 h of PSB0739 or ACSF infusion. Each circle indicates data from one mouse (mean ± s.e.m.; *n* = 6 mice). *P < 0.05 (two-tailed Wilcoxon signed rank test, *P = 0.031). Source data

**Extended Data Fig. 1:**
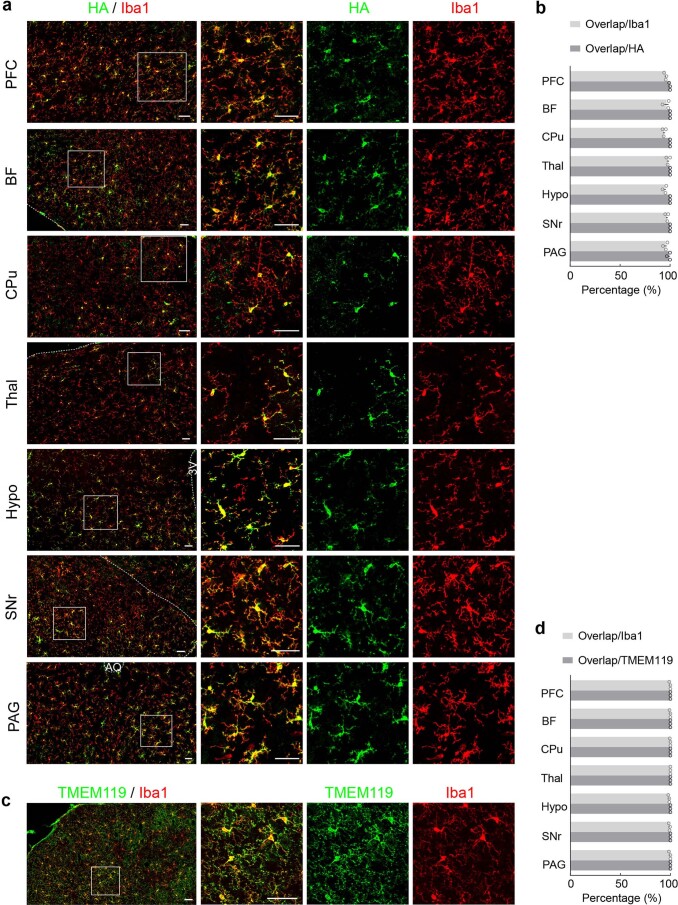
Specificity and efficiency of Gi-DREADD expression in microglia in *Tmem119-CreERT2; R26-LSL-Gi-DREADD* mice. **a**, Confocal images from multiple brain regions showing hM4Di (Gi-DREADD) expression (detected by an HA-tag antibody) in Iba1+ cells. White box in left panel, region enlarged on the right. Scale bar, 50 μm. PFC, prefrontal cortex; BF, basal forebrain; CPu, caudate putamen; Thal, thalamus; Hypo, hypothalamus; SNr, substantia nigra pars reticulata; PAG, periaqueductal gray. **b**, Quantification of efficiency and specificity (n = 3 mice; quantification in Fig. 1b includes all brain regions shown here). ‘Overlap’ refers to cells expressing both HA and Iba1. **c**, Confocal images from the prefrontal cortex showing TMEM119 and Iba1 expression. Scale bar, 50 μm. **d**, Quantification of TMEM119 and Iba1 colocalization in multiple brain regions (n = 3 mice). ‘Overlap’ refers to cells expressing both TMEM119 and Iba1 (although Iba1 can also label infiltrating macrophages, under the condition of this study it shows ~100% overlap with TMEM119 in brain parenchyma**)**. Source data

**Extended Data Fig. 2:**
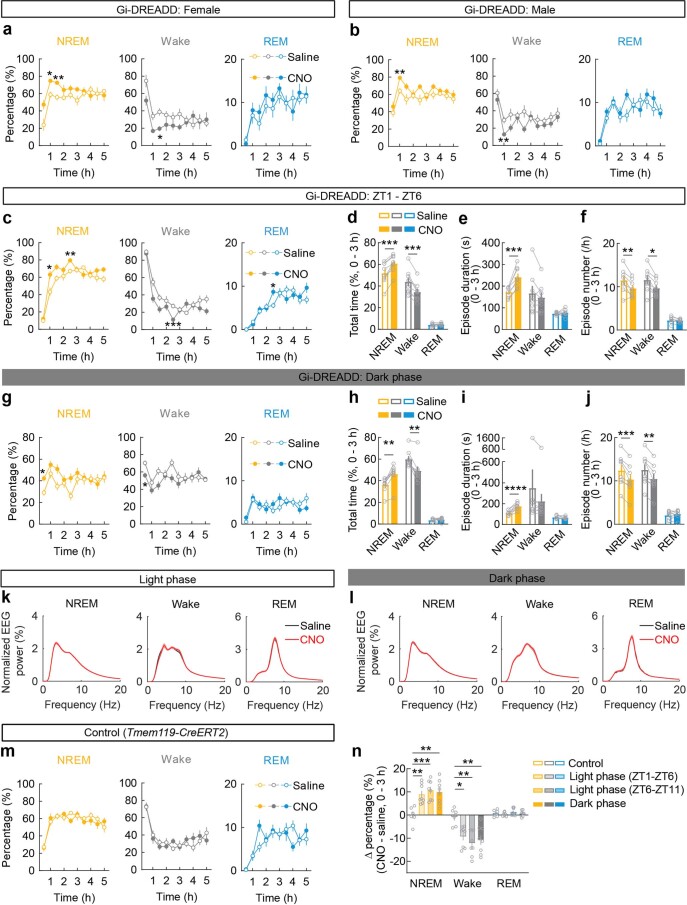
Effect of Gi-DREADD activation on sleep in female and male mice, early light phase, dark phase, EEG power spectra within each state, and control experiments in mice without Gi-DREADD. **a**, **b**, Summary of chemogenetic experiment, analyzed separately for female and male mice. Shown are the percentages of time in each brain state following CNO and saline injection (mean ± s.e.m.; **a**, n = 3 mice; two-way ANOVA with Bonferroni correction; NREM: *P*_*treatment*_ = 0.015; *P*_*time*_ < 0.0001; ^*^P_*1h*_ = 0.017; ^**^P_*1.5h*_ = 0.0089; Wake: *P*_*treatment*_ = 0.016; *P*_*time*_ < 0.0001; ^*^P_*1.5h*_ = 0.015; REM: *P*_*treatment*_ = 0.36; *P*_*time*_ < 0.0001; **b**, n = 5 mice; two-way ANOVA with Bonferroni correction; NREM: *P*_*treatment*_ = 0.0001; *P*_*time*_ < 0.0001; ^**^P_*1h*_ = 0.0057; Wake: *P*_*treatment*_ = 0.0002; *P*_*time*_ < 0.0001; ^**^P_*1h*_ = 0.0092; REM: *P*_*treatment*_ = 0.21; *P*_*time*_ < 0.0001). **c**, Summary of chemogenetic experiment performed between Zeitgeber time (ZT) 1 (8:00 am) and ZT6 (1:00 pm) (mean ± s.e.m.; n = 8 mice: 4 female and 4 male; two-way ANOVA with Bonferroni correction; NREM: *P*_*treatment*_ = 0.0004; *P*_*time*_ < 0.0001; ^*^P_*1h*_ = 0.032; ^**^P_*2.5h*_ = 0.0014; Wake: *P*_*treatment*_ = 0.0007; *P*_*time*_ < 0.0001; ^***^P_*2.5h*_ = 0.0002; REM: *P*_*treatment*_ = 0.40; *P*_*time*_ < 0.0001; ^*^P_*2.5h*_ = 0.047). **d**, Percentage of time in each brain state within 3 h after CNO or saline injection. Each circle indicates data from one mouse (mean ± s.e.m.; n = 8 mice). ^***^P < 0.001 (paired two-tailed *t*-test; ^***^P_*NREM*_ = 0.0005; ^***^P_*Wake*_ = 0.0006). **e**, **f**, Mean episode duration (**e**) and episode number per hour (**f**) for each brain state within 3 h after CNO or saline injection. Each circle indicates data from one mouse (mean ± s.e.m.; n = 8 mice). ^*^P < 0.05, ^**^P < 0.01, ^***^P < 0.001 (paired two-tailed *t*-test; **e**, ^***^P_*NREM*_ = 0.0003; **f**, ^**^P_*NREM*_ = 0.0099; ^*^P_*Wake*_ = 0.010). **g**, Summary of chemogenetic experiment in dark phase (beginning at ~8:00 pm). Shown are the percentages of time in each brain state following CNO and saline injection (mean ± s.e.m.; n = 8 mice; two-way ANOVA with Bonferroni correction; NREM: *P*_*treatment*_ = 0.020; *P*_*time*_ = 0.012; ^*^P_*0.5h*_ = 0.016; Wake: *P*_*treatment*_ = 0.040; *P*_*time*_ = 0.0039; ^*^P_*0.5h*_ = 0.014; REM: *P*_*treatment*_ = 0.89; *P*_*time*_ < 0.0001). **h**, Percentage of time in each brain state within 3 h after CNO or saline injection. Each circle indicates data from one mouse (mean ± s.e.m.; n = 8 mice). ^**^P < 0.01 (two-tailed Wilcoxon signed rank test; ^**^P_*NREM*_ = 0.0078; ^**^P_*Wake*_ = 0.0078). **i**, **j**, Mean episode duration (**i**) and episode number per hour (**j**) for each brain state within 3 h after CNO or saline injection. Each circle indicates data from one mouse (mean ± s.e.m.; n = 8 mice). ^**^P < 0.01, ^***^P < 0.001, ^****^P < 0.0001 (**i**, paired two-tailed *t*-test, ^****^P < 0.0001; **j**, paired two-tailed *t*-test; ^***^P_*NREM*_ = 0.0010; ^***^P_*Wake*_ = 0.0012). **k**, **l**, Comparison of normalized EEG power spectra within each brain state between saline and CNO sessions during the light phase (**k**) or dark phase (**l**), averaged across 8 mice. Shading, ± s.e.m. The EEG spectra of each session was normalized by the total power between 0 and 25 Hz before averaging. **m**, Percentages of time in each brain state following CNO or saline injection in *Tmem119-CreERT2* control mice without Gi-DREADD expression (mean ± s.e.m.; n = 6 mice). **n**, Changes in each brain state induced by chemogenetic manipulation (difference between CNO and saline injections, averaged across 3-h after injection) in mice without Gi-DREADD (control) or with Gi-DREADD (treatment during light phase (ZT1 – ZT6 and ZT6 – ZT11) or dark phase). Each circle indicates data from one mouse (Control, n = 6 mice; ZT1-ZT6, n = 8 mice; ZT6-ZT11, n = 8 mice; dark phase, n = 8 mice); error bar: ± s.e.m. ^*^P < 0.05, ^**^P < 0.01, ^***^P < 0.001 (One-way ANOVA with Holm-Šídák’s test; NREM, *P* = 0.0005; ^**^P_*ZT1-ZT6*_ = 0.0034; ^***^P_*ZT6-ZT11*_ = 0.0007; ^**^P_*dark*_ = 0.0013; Wake, *P* = 0.002; ^*^P_*ZT1-ZT6*_ = 0.019; ^**^P_*ZT6-ZT11*_ = 0.0020; ^**^P_*dark*_ = 0.0064; REM, *P* = 0.49). Source data

To test whether activation of G_i_ signaling in microglia affects sleep, we crossed *Tmem119-CreERT2* driver mice^26^ with reporter mice carrying Cre-inducible hM4Di G_i_–DREADD (a designer receptor exclusively activated by designer drugs), which allowed specific expression of hM4Di in microglia (Fig. 1a–c and Extended Data Fig. 1). Sleep–wake states were measured in freely moving mice in their home cage, and wake and sleep states were classified on the basis of electroencephalogram (EEG) and electromyogram (EMG) recordings. Compared to the control experiment with saline injection, chemogenetic activation of microglia G_i_ signaling induced by intraperitoneal (i.p.) injection of clozapine *N*-oxide (CNO; 1 mg kg^−1^) caused a significant increase in non-rapid eye movement (NREM) sleep and decrease in wakefulness during both light and dark phases (Fig. 1d,e and Extended Data Fig. 2a–d,g,h), primarily due to an increase in the mean duration of NREM sleep episodes (Fig. 1f,g and Extended Data Fig. 2e,f,i–l). In control mice expressing CreERT2 but not G_i_–DREADD, CNO had no significant effect (Extended Data Fig. 2m,n).

**Extended Data Fig. 3:**
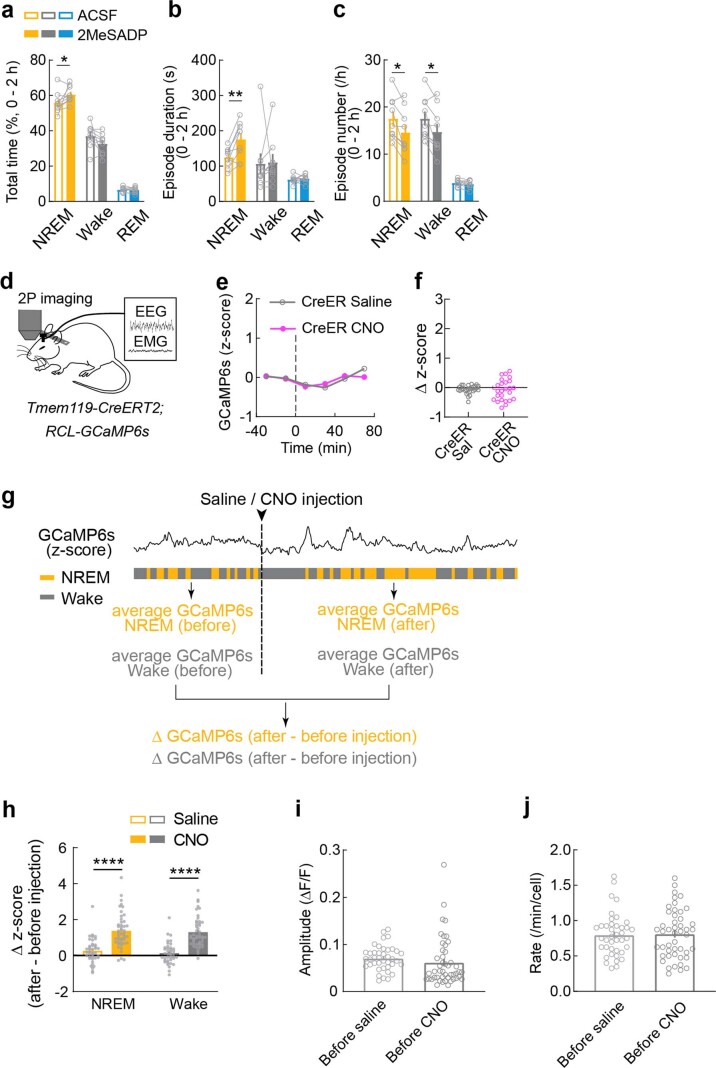
Effect of P2Y12 agonist on sleep, control experiments for Ca^2+^ imaging in mice without Gi-DREADD, comparison of microglia Ca^2+^ activity before and after CNO-induced Gi activation within each brain state, and Ca^2+^ activity during baseline period. **a**-**c**, Percentage of time (**a**), mean episode duration (**b**), and episode number per hour (**c**) for each brain state within 2 h after i.c.v. infusion of P2Y12 agonist (2MeSADP) or ACSF. Each circle indicates data from one mouse; error bar: ± s.e.m. (n = 9 mice). ^*^P < 0.05, ^**^P < 0.01 (**a**, two-tailed Wilcoxon signed rank test, ^*^P = 0.039; **b**, paired two-tailed *t*-test, ^**^P = 0.0030; **c**, paired two-tailed *t*-test; NREM, ^*^P = 0.012; Wake, ^*^P = 0.015). **d**, Schematic for two-photon (2 P) Ca^2+^ imaging in head-fixed mice without Gi-DREADD expression. **e**, Z-scored Ca^2+^ activity averaged across all microglia (CNO: n = 25; saline: n = 28; from 4 mice). Dashed line, time of injection. **f**, Population summary of the change in mean Ca^2+^ level. **g**, Diagram illustrating the comparison of Ca^2+^ activity before and after CNO or saline injection within each brain state. First, Ca^2+^ activity in NREM or wakeful episodes are averaged separately for before and after injection; second, the difference between the averaged Ca^2+^ activity before and after injection are calculated for each brain state. **h**, CNO-induced change in microglia Ca^2+^ for each brain state (difference between before and after injection). CNO, n = 47; saline: n = 40; from 5 mice. Bars, mean ± s.e.m. (two-tailed Mann-Whitney *U* test; NREM, ^****^P < 0.0001; Wake, ^****^P < 0.0001). **i**, **j**, Amplitude (**i**) and frequency (**j**) of Ca^2+^ events during baseline period (before saline or CNO injection). CNO: n = 47; saline: n = 40; from 5 mice. Data are presented as mean ± s.e.m. Source data

Next, to test whether endogenous G_i_ signaling mediated by P2Y12 receptors has a role in sleep–wake regulation, we performed intracerebroventricular (i.c.v.) infusion of PSB0739 (1 mM, 2 μl), a selective P2Y12 receptor antagonist^23^ (Fig. 1h–j). Compared to the control experiment with artificial cerebrospinal fluid (ACSF) infusion, PSB0739 caused a significant decrease in NREM sleep and increase in wakefulness, due mainly to a decrease in NREM episode duration (Fig. 1k–m). In contrast, activation of P2Y12 receptors with i.c.v. infusion of their agonist 2MeSADP (300 mM, 2 μl) increased the episode duration of NREM sleep (Extended Data Fig. 3a–c). Thus, P2Y12 receptor signaling contributes significantly to NREM sleep, especially its maintenance.

### G_i_ activation increases microglia Ca^2+^ activity

In addition to a decrease in cyclic AMP (cAMP) that is expected from the canonical pathway, G_i_ activation can cause an increase in intracellular Ca^2+^ in microglia in vitro^27^. Although Ca^2+^ signaling has been shown to be involved in P2Y12-mediated chemotaxis in vitro^28^, its function in vivo is only beginning to be investigated^29,30^.

**Fig. 2:**
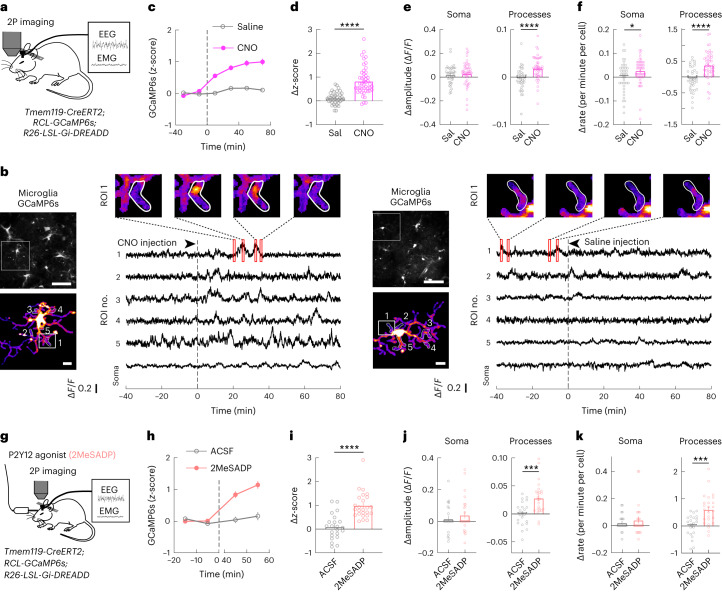
Activation of microglia G_i_ signaling increases intracellular Ca^2+^. **a**, Schematic for two-photon (2P) Ca^2+^ imaging in head-fixed mice. **b**, Example imaging sessions with CNO and saline injection. Top left, field of view (scale bar, 50 μm); bottom left, high-magnification view of the microglia soma and processes in the white box (scale bar, 10 μm); five regions of interest (ROIs) in microglia processes are outlined, whose Ca^2+^ traces are shown on the right, with snapshots of Ca^2+^ transients shown on top (red box and associated dashed line indicate the time period for each snapshot). The dashed line indicates the time of CNO or saline injection. **c**, *z*-scored Ca^2+^ activity averaged across all microglia (CNO, *n* = 47; saline, *n* = 40; from 5 mice: 3 female and 2 male; circles and error bars, mean ± s.e.m.). Dashed line, time of injection. **d**–**f**, Population summary of the CNO-induced change in mean Ca^2+^ level (**d**) and the amplitude (**e**) and frequency (**f**) of Ca^2+^ events in soma and processes (difference between before and after injection). Each circle indicates data from one cell. CNO: somas, *n* = 47; processes, *n* = 1,178; saline (Sal): somas, *n* = 40; processes, *n* = 931. Data are presented as the mean ± s.e.m.; *P < 0.05, ****P < 0.0001 (two-tailed Mann–Whitney *U* test; **d**, ****P < 0.0001; **e**, ****P < 0.0001; **f**, *P = 0.049, ****P < 0.0001). **g**, Schematic for two-photon Ca^2+^ imaging with local application of P2Y12 agonist (2MeSADP). **h**–**k**, Similar to **c**–**f**, but for local infusion of 2MeSADP or ACSF (2MeSADP, *n* = 25; ACSF, *n* = 22; from 5 mice: 3 female and 2 male). Dashed line, time of drug application. Each circle indicates data from one cell. 2MeSADP: somas, *n* = 25; processes, *n* = 581; ACSF: somas, *n* = 22; processes, *n* = 469. Data are shown as mean ± s.e.m.; ***P < 0.001, ****P < 0.0001 (**i**, two-tailed Mann–Whitney *U* test, ****P < 0.0001; **j**, unpaired two-tailed *t*-test, ***P = 0.0003; **k**, unpaired two-tailed *t*-test, ***P = 0.0001). Source data

To measure Ca^2+^ activity following G_i_–DREADD activation, we performed two-photon imaging in the prefrontal cortex of mice expressing both G_i_–DREADD and GCaMP6s specifically in microglia (*Tmem119-CreERT2*; *RCL-GcaMP6s*; *R26-LSL-Gi-DREADD*) (Fig. 2a,b). We chose the prefrontal cortex for its easy accessibility for two-photon imaging and its likely involvement in brain-state regulation through its reciprocal connections with multiple wake-promoting neuromodulatory centers^31,32^. Under the baseline condition, we observed infrequent Ca^2+^ transients (Fig. 2b), consistent with previous studies^29,30^. However, CNO-induced G_i_ activation caused a strong increase in Ca^2+^ activity, primarily in microglia processes. As shown in the population average of GCaMP6s fluorescence, CNO injection caused a progressive Ca^2+^ increase over a period of tens of minutes, whereas saline injection had no significant effect (Fig. 2c and Supplementary Video 1). The increase in Ca^2+^ induced by i.p. injection was significantly higher for CNO than saline in mice expressing G_i_–DREADD in microglia (Fig. 2d; *P* < 0.0001), but not in control mice without G_i_–DREADD (Extended Data Fig. 3d–f; *P* = 0.73). Note, however, that CNO-induced G_i_ activation also increased NREM sleep (Fig. 1a–g), which could in principle contribute to microglia Ca^2+^ increase separately from the direct effect of G_i_ activation. When we compared Ca^2+^ activity before and after i.p. injection within the same brain state (NREM before with NREM after; wake before with wake after), the effect of CNO was still highly significant (Extended Data Fig. 3g,h), indicating that the Ca^2+^ increase was not merely an indirect effect of brain-state changes. In addition to the overall fluorescence, we used a threshold-based method to detect discrete Ca^2+^ events and quantified their amplitudes and frequency^30^. Compared to saline control, CNO injection caused significant increases in both the amplitude and the frequency of Ca^2+^ events, with stronger effects in microglia processes than the soma (Fig. 2e,f and Extended Data Fig. 3i,j). In addition to chemogenetic activation of G_i_ signaling, local application of the P2Y12 agonist 2MeSADP (10 μM, 2 μl) in the prefrontal cortex caused a similar increase in microglia Ca^2+^ activity (Fig. 2g–k), although 2MeSADP may also activate P2Y1 and P2Y13, which could exert synergistic or distinct influences on microglia function^33,34^.

### Role of microglia Ca^2+^ in sleep regulation

**Fig. 3:**
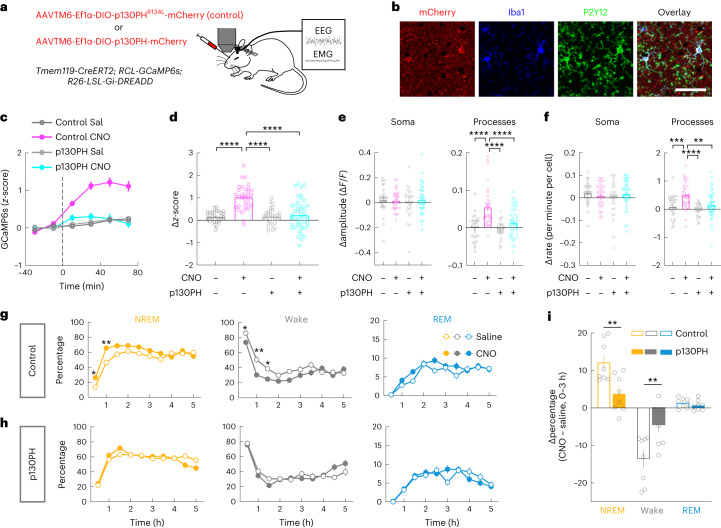
Effect of microglia G_i_ activation on sleep depends on increased intracellular Ca^2+^. **a**, Schematic for two-photon Ca^2+^ imaging with p130PH or p130PH^R134L^ expression. **b**, Confocal images of p130PH–mCherry expression in Iba1^+^P2Y12^+^ microglia in the prefrontal cortex. Scale bar, 50 μm. **c**, *z*-scored Ca^2+^ activity averaged across all microglia imaged from mice expressing p130PH (CNO, *n* = 44; saline, *n* = 31; from 5 mice: 3 female and 2 male) or p130PH^R134L^ (CNO, *n* = 32; saline, *n* = 29; from 5 mice: 2 female and 3 male). Dashed line, time of injection. **d**–**f**, Population summary of the CNO-induced change in mean Ca^2+^ level (**d**) and the amplitude (**e**) and frequency (**f**) of Ca^2+^ events in soma and processes (difference between before and after injection) in mice expressing p130PH or p130PH^R134L^. Each circle indicates data from one cell. p130PH_CNO_: *n* = 44 somas, *n* = 789 processes; p130PH_saline_: *n* = 31 somas, *n* = 457 processes; p130PH^R134L^_CNO_: *n* = 32 somas, *n* = 595 processes; p130PH^R134L^_saline_: *n* = 29 somas, *n* = 564 processes. **P < 0.01, ****P < 0.0001 (one-way ANOVA with Holm–Šídák’s test; **d**, *P* < 0.0001, ****P < 0.0001; **e**, *P*_soma_ = 0.65, *P*_processes_ < 0.0001, ****P < 0.0001; **f**, *P*_soma_ = 0.50, *P*_processes_ < 0.0001, ***P = 0.0007, ****P < 0.0001, **P = 0.0024). **g**,**h**, Effect of microglia G_i_ activation on sleep in mice expressing p130PH^R134L^ (**g**; *n* = 8 mice: 4 female and 4 male) or p130PH (**h**; *n* = 8 mice: 3 female and 5 male). *P < 0.05, **P < 0.01 (two-way ANOVA with Bonferroni correction; p130PH^R134L^: NREM: *P*_treatment_ < 0.0001, *P*_time_ < 0.0001, *P_0.5h_ = 0.038, **P_1h_ = 0.0036; wake: *P*_treatment_ = 0.0002, *P*_time_ < 0.0001, *P_0.5h_ = 0.044, **P_1h_ = 0.0051, *P_1.5h_ = 0.019; REM: *P*_treatment_ = 0.036, *P*_time_ < 0.0001; p130PH: NREM: *P*_treatment_ = 0.94, *P*_time_ < 0.0001; wake: *P*_treatment_ = 0.95, *P*_time_ < 0.0001; REM: *P*_treatment_ = 0.97, *P*_time_ < 0.0001). **i**, Changes in each brain state induced by chemogenetic activation (difference between CNO and saline injections, averaged across 3 h after injection) in mice expressing p130PH^R134L^ or p130PH. Each circle indicates data from one mouse (p130PH^R134L^, *n* = 8 mice; p130PH, *n* = 8 mice). **P < 0.01 (unpaired two-tailed *t*-test, **P_NREM_ = 0.0055, **P_wake_ = 0.0082). Data are presented as the mean ± s.e.m. Source data

**Extended Data Fig. 4:**
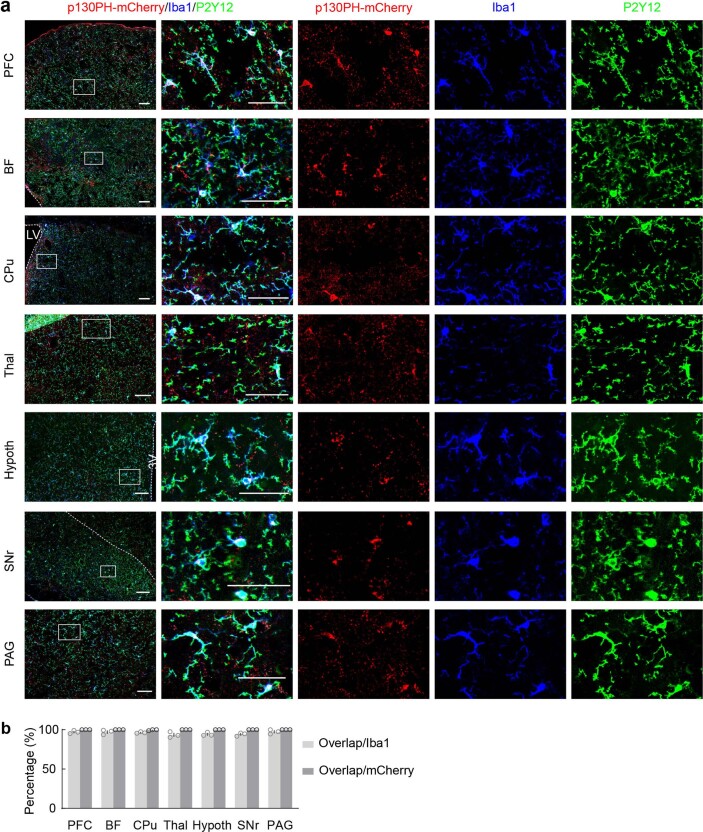
Efficiency and specificity of p130PH-mCherry expression in microglia. **a**, Confocal images of p130PH-mCherry expression in Iba1 + , P2Y12+ microglia in multiple brain regions. White box in left panel, region enlarged on the right. Scale bar: left, 100 μm; right, 50 μm. PFC, prefrontal cortex; BF, basal forebrain; CPu, caudate putamen; Thal, thalamus; Hypo, hypothalamus; SNr, substantia nigra pars reticulata; PAG, periaqueductal gray. **b**, Quantification of efficiency and specificity. Bars, mean ± s.e.m. (n = 3 mice). ‘Overlap’ refers to cells expressing both p130PH-mCherry and Iba1. Source data

**Extended Data Fig. 5:**
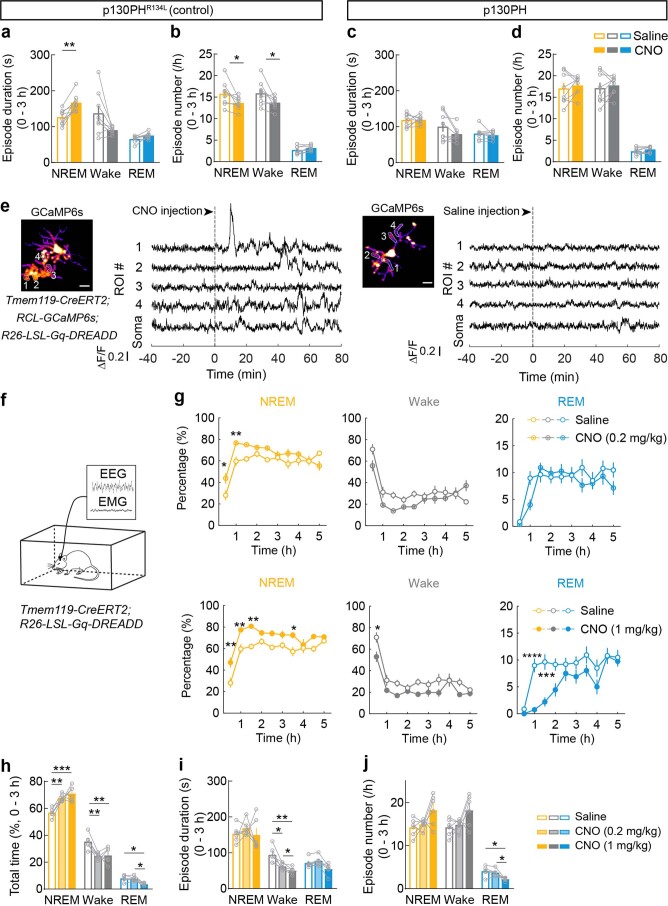
Effects of Gi-DREADD activation in mice expressing p130PH^R134L^ or p130PH and effect of Gq-DREADD activation on microglia Ca^2+^ and sleep. **a**, **b**, Mean episode duration (**a**) and episode number per hour (**b**) for each brain state within 3 h after CNO or saline injection in mice expressing p130PH^R134L^. Each circle indicates data from one mouse; bars: mean ± s.e.m. (n = 8 mice). ^*^P < 0.05, ^**^P < 0.01 (paired two-tailed *t*-test; **a**, ^**^P = 0.0021; **b**, ^*^P_*NREM*_ = 0.045; ^*^P_*Wake*_ = 0.046). **c**, **d**, Similar to (**a**, **b**), but for mice expressing p130PH (n = 8 mice). **e**, Example imaging sessions with CNO and saline injection in mice expressing GCaMP6s and Gq-DREADD in microglia. Left, representative microglia (scale bar, 10 μm); 4 ROIs in microglia processes are outlined, whose Ca^2+^ traces are shown on the right. Dashed line, time of injection. **f**, Schematic for chemogenetic experiment with Gq-DREADD mice. **g**, Percentages of time in each brain state following CNO or saline injection (top, CNO: 0.2 mg/kg; bottom, CNO: 1 mg/kg; mean ± s.e.m.; n = 6 mice). ^*^P < 0.05, ^**^P < 0.01, ^***^P < 0.001, ^****^P < 0.0001 (two-way ANOVA with Bonferroni correction; top, NREM: *P*_*treatment*_ < 0.0001; *P*_*time*_ < 0.0001; ^*^P_*0.5h*_ = 0.015; ^**^P_*1h*_ = 0.0063; Wake: *P*_*treatment*_ = 0.0043; *P*_*time*_ < 0.0001; REM: *P*_*treatment*_ = 0.78; *P*_*time*_ < 0.0001; bottom, NREM: *P*_*treatment*_ < 0.0001; *P*_*time*_ < 0.0001; ^**^P_*0.5h*_ = 0.0011; ^**^P_*1h*_ = 0.0031; ^**^P_*1.5h*_ = 0.0012; ^*^P_*3.5h*_ = 0.019; Wake: *P*_*treatment*_ = 0.0002; *P*_*time*_ < 0.0001; ^*^P_*0.5h*_ = 0.012; REM: *P*_*treatment*_ = 0.0002; *P*_*time*_ < 0.0001; ^****^P_*1h*_ < 0.0001; ^***^P_*1.5h*_ = 0.0002). **h-j**, Percentage of time (**h**), mean episode duration (**i**) and episode number per hour (**j**) for each brain state within 3 h after CNO or saline injection. Each circle indicates data from one mouse, bars: mean ± s.e.m. (n = 6 mice). ^*^P < 0.05, ^**^P < 0.01, ^***^P < 0.001 (One-way ANOVA with Holm-Šídák’s test; **h**, NREM, *P* = 0.0029; ^**^P = 0.0025; ^***^P = 0.0009; Wake, *P* = 0.0037; ^**^P_*0.2mg/kg*_ = 0.0072; ^**^P_*1mg/kg*_ = 0.0039; REM, *P* = 0.0011; ^*^P_*1mg/kg*_ = 0.011; ^*^P_*0.2 vs. 1mg/kg*_ = 0.011; **i**, NREM, *P* = 0.55; Wake, *P* = 0.003; ^*^P_*0.2mg/kg*_ = 0.028; ^**^P_*1mg/kg*_ = 0.0025; ^*^ *P*_*0.2 vs. 1mg/kg*_ = 0.028; REM, *P* = 0.028; **j**, NREM, *P* = 0.074; Wake, *P* = 0.083; REM, *P* = 0.013; ^*^P_*1mg/kg*_ = 0.041; ^**^P_*0.2 vs. 1mg/kg*_ = 0.041). Source data

We next tested the causal role of microglia Ca^2+^ activity in regulating sleep. A previous in vitro study implicated the phospholipase C (PLC)–inositol 1,4,5-trisphosphate (IP_3_)–Ca^2+^ cascade in P2Y12-mediated microglia chemotaxis^28^. We expressed the pleckstrin homology domain of PLC-like protein p130 (p130PH), which buffers cytosolic IP_3_ to inhibit Ca^2+^ release from the internal store^35,36^. AAVTM6 (AAV6 with a triple mutation that transduces microglia more efficiently^37^) with Cre-dependent expression of either p130PH or p130PH^R134L^ (a mutated form of p130PH that does not bind to IP_3_) was injected into *Tmem119-CreERT2;* *RCL-GCaMP6s;* *R26-LSL-Gi-DREADD* mice (Fig. 3a,b and Extended Data Fig. 4). While, in control mice (expressing p130PH^R134L^), CNO-induced G_i_ activation caused a significant Ca^2+^ elevation, no significant change was observed in mice expressing p130PH; the effect of G_i_ activation on Ca^2+^ activity was significantly different between p130PH and p130PH^R134L^ mice (Fig. 3c–f). Importantly, the effect of G_i_ activation on sleep was also largely abolished in mice with p130PH but not p130PH^R134L^ (Fig. 3g–i and Extended Data Fig. 5a–d). This suggests that microglia Ca^2+^ activity is necessary for the sleep-promoting effect.

In addition to suppressing the G_i_-induced increase in Ca^2+^ activity, we elevated microglia Ca^2+^ through G_q_-mediated activation of PLC–IP_3_ signaling. In mice expressing hM3Dq (G_q_–DREADD) specifically in microglia (*Tmem119-CreERT2*; *R26-LSL-Gq-DREADD*), CNO-induced G_q_ activation significantly increased NREM sleep, with a magnitude comparable to that caused by G_i_ activation (Extended Data Fig. 5e–j). Together, these results indicate that microglia Ca^2+^ activity has an important role in sleep regulation.

### Microglia Ca^2+^ activity across natural sleep–wake states

**Fig. 4:**
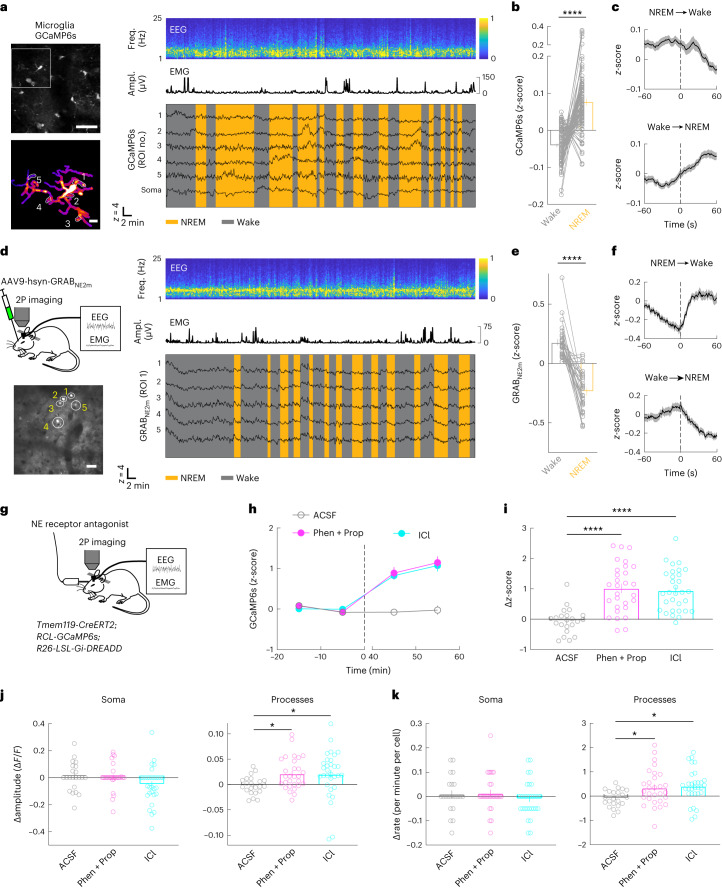
Modulation of microglia Ca^2+^ by brain state and NE. **a**, An example Ca^2+^ imaging session. Top left, field of view containing multiple microglia (scale bar, 50 μm); bottom left, high-magnification view of the microglia soma and processes in the white box (scale bar, 10 μm); five ROIs in processes are outlined, whose Ca^2+^ traces are shown on the right together with the EEG spectrogram (normalized by the maximum of each session), EMG amplitude and brain states. **b**, Summary of microglia Ca^2+^ activity during wake and NREM states. Each line presents data from one cell (*n* = 87 cells, from 5 mice: 3 female and 2 male; two-tailed Wilcoxon signed rank test, ****P < 0.0001). **c**, Ca^2+^ activity at brain-state transitions (*n* = 87 cells). Dashed line, time of transition; shading, ±s.e.m. **d**, Imaging of NE signals. Top left, schematic for two-photon imaging of GRAB_NE2m_ fluorescence in the prefrontal cortex; bottom left, field of view (scale bar, 50 μm); right, NE traces of ROIs indicated in bottom-left image. **e**, Average NE signals in wake and NREM states. Each line represents data from one session (*n* = 30 sessions, from 14 mice: 6 female and 8 male; a total of 8–12 ROIs were assessed for each session; two-tailed Wilcoxon signed rank test, ****P < 0.0001). **f**, Similar to **c**, but for NE signals (averaged across 30 sessions). **g**, Schematic of microglia Ca^2+^ imaging with local application of NE receptor antagonist. **h**, Microglia Ca^2+^ before and after application of ICl (β_2_ receptor antagonist), phentolamine (Phen; α receptor antagonist) and propranolol (Prop; β receptor antagonist), or ACSF, averaged across 31 (ICl), 29 (Phen + Prop) or 22 (ACSF) cells from 5 mice: 3 female and 2 male. Dashed line, time of drug application. **i**–**k**, Difference in mean Ca^2+^ level (**i**) and the amplitude (**j**) and frequency (**k**) of Ca^2+^ events before and after drug application. Each circle indicates data from one cell. ICl: *n* = 31 somas, *n* = 660 processes; Phen + Prop: *n* = 29 somas, *n* = 674 processes; ACSF: *n* = 22 somas; *n* = 441 processes. *P < 0.05, ****P < 0.0001 (one-way ANOVA with Holm–Šídák’s test; **i**, *P* < 0.0001, ****P < 0.0001; **j**, *P*_soma_ = 0.10, *P*_processes_ = 0.045, *P_Phen+Prop_ = 0.0495, *P_ICl_ = 0.0495; **k**, *P*_soma_ = 0.44, *P*_processes_ = 0.014, *P_Phen+Prop_ = 0.018, *P_ICl_ = 0.011). Data are presented as the mean ± s.e^.^m. Source data

**Extended Data Fig. 6:**
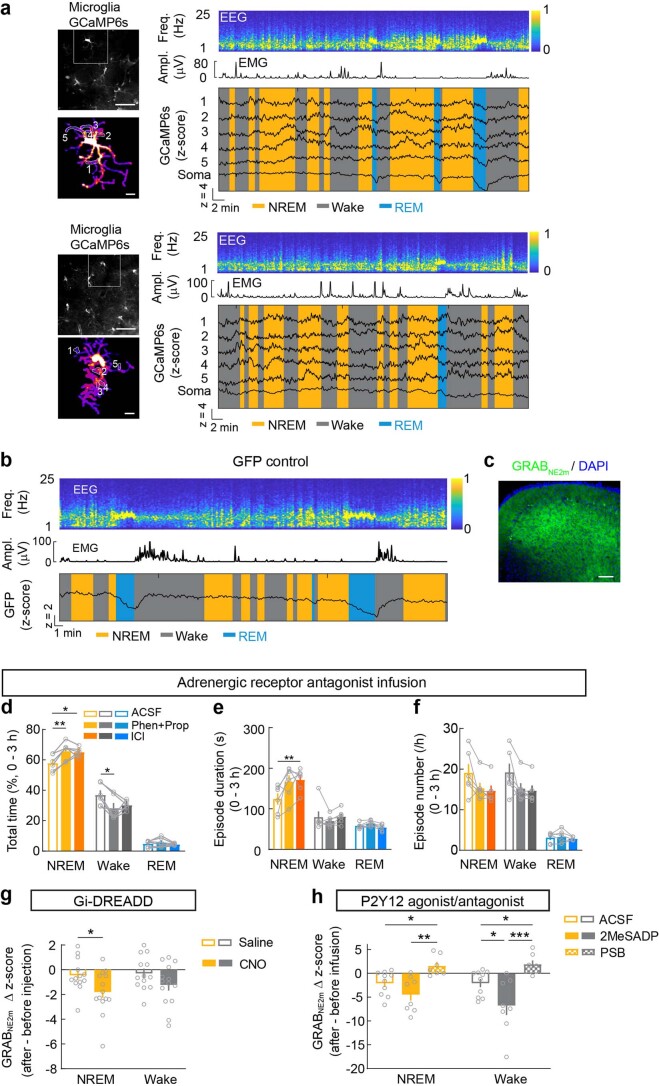
Ca^2+^ imaging sessions with episodes of wakefulness, NREM sleep, and REM sleep, control experiment for 2 P imaging with Ca^2+^-independent GFP, GRAB_NE_ expression, effect of adrenergic receptor antagonists on sleep, and comparison of cortical NE activity before and after microglia manipulations within the same brain state. **a**, Two examples of Ca^2+^ imaging session. Top left, field of view containing multiple microglia (scale bar, 50 μm); Bottom left, high magnification view of the microglia soma and processes in the white box (scale bar, 10 μm); 5 ROIs in processes are outlined, whose Ca^2+^ traces are shown on the right together with EEG spectrogram (Freq., frequency), EMG amplitude (Ampl.), and color-coded brain states. **b**, An example 2 P imaging session with GFP showing consistent decrease in fluorescence during REM sleep. Top, EEG spectrogram (Freq., frequency); middle, EMG amplitude (Ampl.); bottom, GFP signal (from a *Cx3cr1*^*eGFP/+*^ mouse) and color-coded brain states. **c**, Image showing the expression of the GRAB_NE2m_ in the prefrontal cortex. Scale bar, 100 μm. **d-f**, Percentage of time (**d**), mean episode duration (**e**), and episode number per hour (**f**) within 3 h after i.c.v. infusion of Phen (α receptor antagonist) and Prop (β receptor antagonist), ICl (β2 receptor antagonist), or ACSF. Each circle indicates data from one mouse; bars, mean ± s.e.m.; n = 6 mice. ^*^P < 0.05, ^**^P < 0.01 (One-way ANOVA with Holm-Šídák’s test; **d**, NREM, *P* = 0.0058; ACSF vs. Phen+Prop, ^**^P = 0.0037; ACSF vs. ICl, ^*^P = 0.017; Wake, *P* = 0.023; ACSF vs. Phen+Prop, ^*^P = 0.010; REM, *P* = 0.37; **e**, NREM, *P* = 0.037; ACSF vs. ICl, ^**^P = 0.0012; Wake, *P* = 0.42; REM, *P* = 0.18; **f**, NREM, *P* = 0.053; Wake, *P* = 0.048; REM, *P* = 0.72;). **g**, Change of NE activity in each brain state induced by chemogenetic activation of microglia Gi signaling (difference between before (-40 – 0 min) and after (0 – 120 min) injection) (mean ± s.e.m., saline, n = 13 sessions, CNO, n = 14; from 5 mice; 8 to 12 ROIs were assessed for each session). ^*^P < 0.05 (unpaired two-tailed *t*-test, ^*^P = 0.033). **h**, Similar to (**g**), but for local perfusion experiments (difference between before (-20 – 0 min) and after (40 min – 60 min) drug perfusion) (mean ± s.e.m.; 2MeSADP, n = 8 sessions; PSB, n = 7; ACSF, n = 10, from 4 mice; 8 to 12 ROIs were assessed for each session). ^*^P < 0.05, ^**^P < 0.01, ^***^P < 0.001 (One-way ANOVA with Holm-Šídák’s test; NREM, *P* = 0.002; ACSF vs. PSB, ^*^P = 0.034; 2MeSADP vs. PSB, ^**^P = 0.0015; Wake, *P* = 0.001; ACSF vs. 2MeSADP, ^*^P = 0.033; ACSF vs. PSB, ^*^P = 0.046; 2MeSADP vs. PSB, ^***^P = 0.0007). Source data

We next examined whether microglia Ca^2+^ levels naturally change between sleep and wake states. After extensive habituation, mice exhibited multiple episodes of wakefulness and NREM and REM sleep during each imaging session under the head-fixed condition^38^ (Extended Data Fig. 6a). Because, during REM sleep, fluorescence imaging may be confounded by non-Ca^2+^-related changes such as in metabolic rate and blood flow^39^ (Extended Data Fig. 6b), we focused on NREM sleep and wakeful states. We observed a significant decrease in microglia Ca^2+^ at NREM→wake transitions and an increase at wake→NREM transitions, resulting in a higher level of Ca^2+^ activity during NREM sleep than wakefulness (Fig. 4a–c). Together with the observation that G_i_-induced Ca^2+^ increase promoted NREM sleep, this suggests that endogenous microglia Ca^2+^ activity both regulates and is regulated by sleep–wake states.

One of the most important wake-promoting neuromodulators is NE^40,41^, which also powerfully modulates microglia motility^42,43^. We wondered whether NE has a role in regulating microglia Ca^2+^ activity across brain states. Imaging in the prefrontal cortex of mice expressing a GPCR-activation-based fluorescent NE sensor (GRAB_NE_)^44^ showed a significant increase in cortical NE level at NREM→wake transitions and decrease at wake→NREM transitions (Fig. 4d–f and Extended Data Fig. 6c), although we observed NE level fluctuations even during NREM sleep. These observations are consistent with recent studies based on fiber photometry recording^45,46^, as well as previous electrophysiological recordings from locus coeruleus neurons^47^, the main source of NE in the mammalian forebrain^48^. However, they are opposite to the changes in microglia Ca^2+^ activity (Fig. 4a–c).

To test whether NE modulates microglia Ca^2+^, we applied adrenergic receptor antagonists, which can increase NREM sleep (Extended Data Fig. 6d–f). Local application of either ICl-118,551 (ICl; 30 μM; a selective β_2_ receptor antagonist) or a combination of phentolamine (50 μM; antagonist to α receptors) and propranolol (10 μM; antagonist to β receptors) in the prefrontal cortex caused a marked increase in microglia Ca^2+^ (Fig. 4g–k), indicating that the reduction in NE signaling during sleep is at least partly responsible for the increased Ca^2+^ activity (Fig. 3a–c). Note, however, that the effect of these antagonists could be mediated either directly by adrenergic receptors on microglia or indirectly through their effects on neurons and astrocytes, which may in turn affect microglia Ca^2+^ activity.

### Activation of microglia G_i_ suppresses NE transmission

**Fig. 5:**
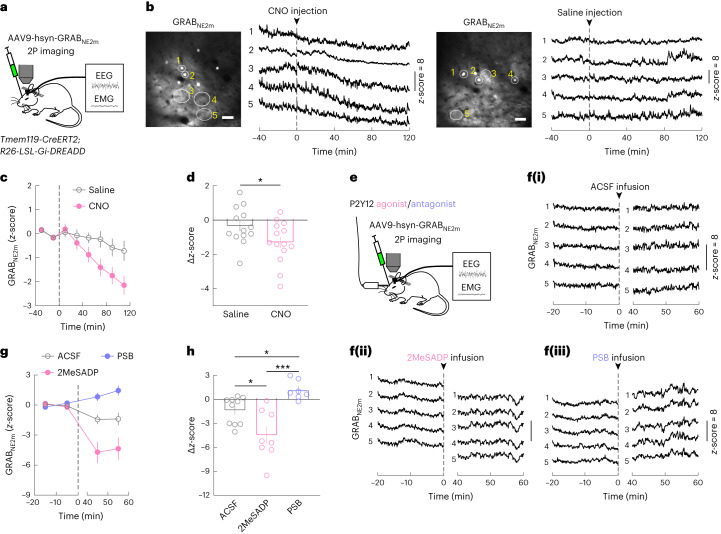
Activation of microglia G_i_ signaling suppresses NE transmission. **a**, Schematic of GRAB_NE2m_ imaging in the prefrontal cortex. **b**, Example imaging session with chemogenetic G_i_ activation in microglia. Left, field of view (scale bar, 50 μm); five ROIs are outlined, whose NE traces are shown on the right. Dashed line, time of CNO or saline injection. **c**, Effect of chemogenetic G_i_ activation in microglia on NE signals averaged across 13 (saline) or 14 (CNO) sessions from 5 mice: 3 female and 2 male. A total of 8–12 ROIs were assessed for each session. The dashed line indicates the time of injection. **d**, Difference in NE before and after (20–120 min) saline or CNO injection. Each circle represents data from one session (saline, *n* = 13 sessions; CNO, *n* = 14 sessions; from 5 mice). Data are presented as the mean ± s.e.m.; *P < 0.05 (unpaired two-tailed *t*-test, *P = 0.041). **e**, Schematic of GRAB_NE2m_ imaging with local application of P2Y12 agonist or antagonist. **f**, NE traces from example imaging sessions with ACSF (**i**), 2MeSADP (**ii**) or PSB0739 (**iii**) application. The dashed line indicates the time of drug application. **g**,**h**, Similar to **c**,**d** for local drug application experiments (**e**); 2MeSADP, *n* = 8 sessions; PSB0739, *n* = 7 sessions; ACSF, *n* = 10 sessions; from 4 mice: 2 female and 2 male. The dashed line indicates the time of drug application. *P < 0.05, ***P < 0.001 (one-way ANOVA with Holm–Šídák’s test, *P* = 0.0002; ACSF versus 2MeSADP, *P = 0.012; ACSF versus PSB0739, *P = 0^.^026; 2MeSADP versus PSB0739, ***P = 0.0001; the Δ*z*-score was computed as the difference between the periods [−20, 0] and [40, 60] min). Data are presented as the mean ± s.e.m. Source data

**Fig. 6:**
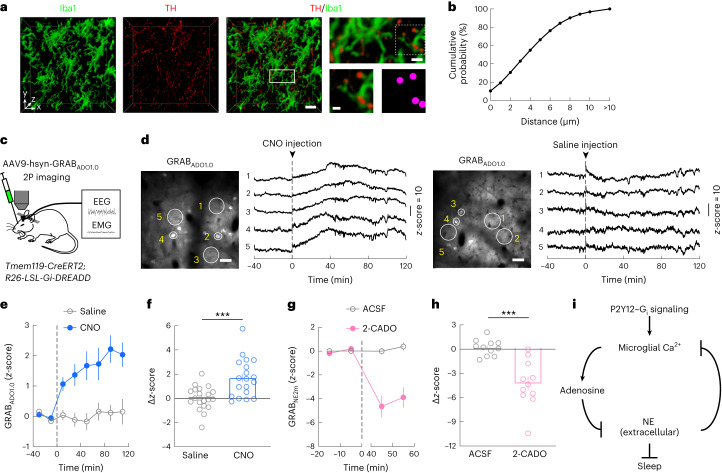
Suppression of NE transmission by microglia G_i_ signaling is partly mediated by elevated adenosine level. **a**, Example images of Iba1-labeled microglia (green; left) and TH-labeled axons (red; middle left) in the prefrontal cortex. Middle right, three-dimensional (3D) rendering image of a 50-μm-thick slice (scale bar, 20 μm); top right, high-magnification view of the boxed region (scale bar, 5 μm); bottom right, further enlarged view of the region in the dashed box and automatically detected axon boutons (magenta) (scale bar, 2 μm). **b**, Distance of boutons to the nearest microglia (*n* = 140,884 boutons from the prefrontal cortex of 3 mice). **c**, Schematic of GRAB_ADO_ imaging in the prefrontal cortex. **d**, Example imaging session with chemogenetic G_i_ activation in microglia. Left, field of view (scale bar, 50 μm); five ROIs are outlined, whose adenosine traces are shown on the right. Dashed line, time of CNO or saline injection. **e**, Effect of chemogenetic G_i_ activation in microglia on adenosine signals averaged across 20 (saline) or 19 (CNO) sessions from 6 mice. A total of 8–12 ROIs were assessed for each session. The dashed line indicates the time of injection. **f**, Difference in adenosine before and after saline or CNO injection. Each circle represents data from one session (saline, *n* = 20 sessions; CNO, *n* = 19 sessions; from 6 mice). Data are presented as the mean ± s.e.m.; ***P < 0.001 (unpaired two-tailed *t*-test, ***P = 0.0005). **g**,**h**, Extracellular NE levels before and after application of 2-CADO (a metabolically stable analog of adenosine). The dashed line indicates the time of application. Each circle indicates data from one session. 2-CADO, *n* = 11 sessions; ACSF, *n* = 11 sessions; from 4 mice. Data are presented as the mean ± s.e.m.; ***P < 0.001 (unpaired two-tailed *t*-test, ***P = 0.0001; the Δ*z*-score was computed as the difference between the periods [−20, 0] and [40, 60] min). **i**, Diagram summarizing microglia regulation of sleep through reciprocal interactions between microglia Ca^2+^ signaling and NE transmission. Data are presented as the mean ± s.e.m. Source data

Knowing the effect of NE on microglia Ca^2+^, we wondered whether microglia Ca^2+^ signaling can in turn regulate NE transmission. In *Tmem119-CreERT2*; *R26-*LSL*-Gi-DREADD* mice expressing GRAB_NE_ in the prefrontal cortex (Fig. 5a), CNO-induced G_i_ activation in microglia caused a strong reduction in cortical extracellular NE concentration (Fig. 5b–d and Extended Data Fig. 6g), indicating a mutually antagonistic relationship between NE transmission and microglia Ca^2+^ signaling (Fig. 6i). Given the powerful role of NE in promoting wakefulness, the sleep-promoting effect of microglia P2Y12–G_i_ signaling is likely mediated at least in part by the reduction in extracellular NE concentration.

**Extended Data Fig. 7:**
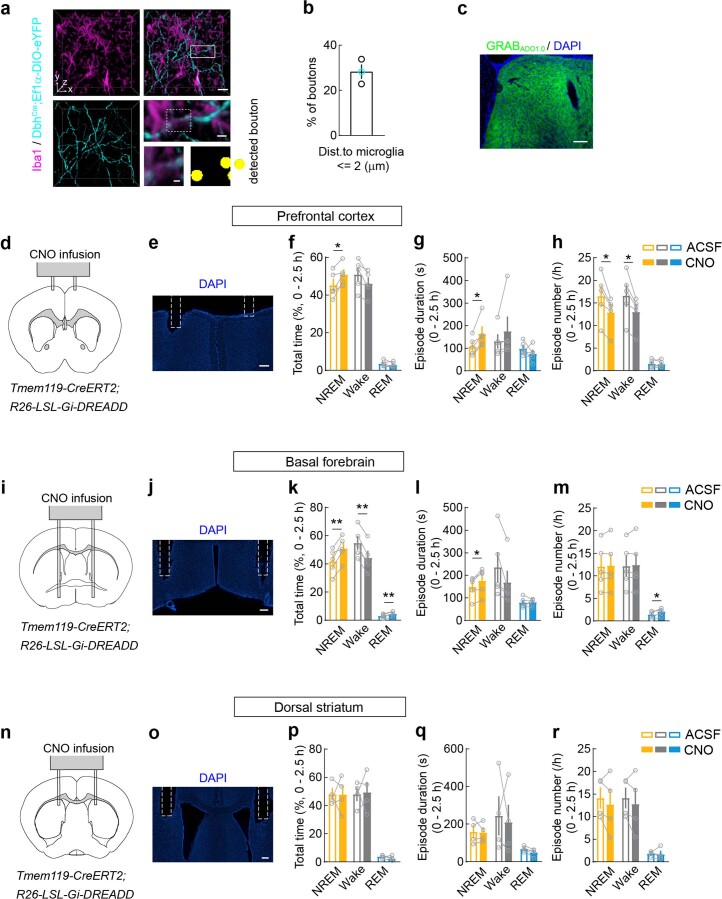
Distance of adrenergic axon boutons to microglia, GRAB_ADO_ expression, and effect of local Gi-DREADD activation on sleep. **a**, Example images of Iba1-labeled microglia (magenta) and Dbh-eYFP-labeled axons (cyan) in prefrontal cortex. Left and top right, 3D rendering images of a 50-μm-thick slice; scale bar, 20 μm; middle right, high-magnification view of the boxed region; scale bar, 5 μm; bottom right, further enlarged view of the region in dashed box and automatically detected axon buttons (yellow); scale bar, 2 μm. **b**, Percentage of buttons within 2 μm from the nearest microglia. Bars, mean ± s.e.m. Each circle represents data from one mouse (n = 3 mice; black circle, TH-labeled axon boutons detected by immunohistochemistry; cyan circle, Dbh-eYFP-labeled boutons). Dist., distance. **c**, Image showing the expression of the GRAB_ADO_ in the prefrontal cortex. Scale bar, 100 μm. **d**, **e**, Coronal diagram (**d**) and image (**e**) showing local infusion site in the prefrontal cortex of *Tmem119-CreERT2; R26-LSL-Gi-DREADD* mice. Scale bar, 200 μm. **f-h**, Percentage of time (**f**), mean episode duration (**g**), and episode number per hour (**h**) for each brain state within 2.5 h after CNO or ACSF infusion. Each circle indicates data from one mouse (mean ± s.e.m.; n = 5 mice). ^*^P < 0.05, ^**^P < 0.01 (**f**, paired two-tailed *t*-test, ^*^P = 0.024; **g**, paired two-tailed *t*-test, ^*^P = 0.022; **h**, paired two-tailed *t*-test; NREM, ^*^P = 0.016; Wake, ^*^P = 0.020). **i-m**, Similar to (**d-h**), but for basal forebrain infusion (n = 5 mice; **k**, paired two-tailed *t*-test; NREM, ^**^P = 0.0067; Wake, ^**^P = 0.0028; REM, ^**^P = 0.0025; **l**, paired two-tailed *t*-test, ^*^P = 0.030; **m**, paired two-tailed *t*-test, ^*^P = 0.020). **n-r**, Similar to (**d-h**), but for dorsal striatum infusion (n = 4 mice). Source data

To test whether manipulation of microglia within the prefrontal cortex is sufficient for NE reduction, we performed local perfusion of P2Y12 receptor agonist or antagonist. Application of the agonist 2MeSADP (10 μM), which increased microglia Ca^2+^ (Fig. 2g–k), also caused a strong decrease in NE signals, whereas the antagonist PSB0739 (250 μM) caused an NE increase (Fig. 5e–h and Extended Data Fig. 6h). Given the large physical separation between the prefrontal cortex and locus coeruleus (the sole source of NE in the cortex^48^), this suggests that microglia can regulate either the release or the reuptake of NE at the axon terminals of locus coeruleus neurons independently of the spiking activity of the cell bodies. Indeed, light-sheet imaging of tyrosine hydroxylase (TH)^+^ axon terminals together with Iba1-labeled microglia confirmed their spatial proximity (Fig. 6a,b and Extended Data Fig. 7a,b), providing ample opportunities for local interactions.

Adenosine is known to inhibit the release of neurotransmitters and neuromodulators, including NE^49^, and microglia can catabolize ATP and ADP to adenosine by the ectonucleotidases CD39 and CD73 (ref. ^22^). We next tested whether activation of microglia G_i_ signaling affects the extracellular adenosine concentration. Imaging in the prefrontal cortex of *Tmem119-CreERT2*; *R26-LSL-Gi-DREADD* mice expressing the adenosine sensor GRAB_ADO_ (ref. ^50^) showed that CNO-induced G_i_ activation in microglia caused a strong increase in adenosine level compared to the control (Fig. 6c–f and Extended Data Fig. 7c). Furthermore, application of 2-chloroadenosine (2-CADO; 1 μM), a metabolically stable analog of adenosine, caused a strong decrease in the NE level (Fig. 6g,h). Together, these results suggest that the suppression of NE transmission by microglia G_i_ activation is at least partly mediated by an increase in the adenosine level (Fig. 6i).

## Discussion

We showed that activation of G_i_ signaling in microglia promotes sleep (Fig. 1) and that the effect is mediated at least partly by their intracellular Ca^2+^ signaling (Figs. 2 and 3), leading to a reduction in extracellular NE concentration (Figs. 5 and 6). Although our imaging experiments were performed in the prefrontal cortex (for its easy accessibility and likely involvement in brain-state regulation), region-specific G_i_ activation through local CNO infusion (which may affect brain tissues within hundreds of micrometers of the infusion site^51^) suggests that multiple brain regions contribute to the sleep-promoting effect, including the basal forebrain (Extended Data Fig. 7d–r). Given the spatial heterogeneity of microglia^5,8,9^, it would be important for future studies to characterize their Ca^2+^ activity in multiple brain regions. Previous studies showed that microglia depletion either increases NREM sleep specifically in the dark phase^15,16^ or has no significant effect on sleep^17^. A major difference between our study and these earlier studies lies in the time scale; whereas our study focused on dynamic changes over minutes to hours, loss of microglia over days to weeks is likely to cause compensatory changes that may affect sleep through different mechanisms. In future studies, it would be interesting to test the effects of microglia depletion on NE and adenosine activity in the brain. In addition to microglia, astrocytes can promote NREM sleep^52–54^. Unlike microglia, however, astrocyte Ca^2+^ activity is higher during wakefulness than during both anesthesia^55^ and sleep^52–54^, and it is elevated by NE^56,57^. One possibility is that the strong elevation of astrocyte Ca^2+^ during prolonged wakefulness^54^ can cause an increase in Ca^2+^-dependent ATP release^58^, which may activate microglia P2Y12–G_i_ signaling to drive sleep.

P2Y12–G_i_ signaling is crucial for directing microglia extension toward active neurons^20,21,23^ and downregulation of neuronal activity^11,22,59,60^. Here, we showed that G_i_ activation suppressed NE release (Fig. 5 and Extended Data Fig. 6g,h), which may be mediated by increased extracellular adenosine (Fig. 6c–h). In addition to increasing adenosine, microglia exhibit Ca^2+^-dependent release of cytokines such as TNFα^61^, which is known to promote NREM sleep^19^. Interestingly, low doses of TNFα were shown to promote NREM sleep without affecting REM sleep, but high doses of TNFα also suppressed REM sleep^62^. In our study, G_q_ activation with a high dose (1 mg kg^−1^) but not low dose (0.2 mg kg^−1^) of CNO caused suppression of REM sleep (Extended Data Fig. 5f–j), perhaps because G_q_ signaling induced stronger cytokine release at the high CNO dosage^63^.

Chronic sleep restriction can induce both morphological and molecular changes in microglia^64^. Recent studies have shown that, during wakefulness, microglia exhibit reduced motility compared to anesthetized states, likely due to suppression by NE^42,43,65^. Here we showed that microglia intracellular Ca^2+^ level changes with brain states (Fig. 4c), which is also mediated at least in part by changed extracellular NE concentration (Fig. 4h–k). In addition to being suppressed by NE (Fig. 4), we showed that microglia Ca^2+^ signaling can in turn cause a rapid reduction in NE (Fig. 5), thus demonstrating a mutually antagonistic relationship between microglia Ca^2+^ and NE transmission in the brain.

Sleep disruption is increasingly recognized as an important risk factor for Alzheimer’s and other neurodegenerative diseases^14^, and the loss of microglia homeostatic functions is associated with both sleep–wake disruption^15,16^ and disease progression^12,13^. Our findings point to a mechanistic explanation: an increase in microglia Ca^2+^ enabled by sleep may allow more efficient surveillance and clearance of harmful extracellular proteins involved in neurodegeneration^3,4^; reciprocally, microglia also actively promote sleep for the maintenance of brain homeostasis.

## Methods

### Animals

The following mice were obtained from Jackson Laboratory (Jackson stock number in parentheses): *Tmem119-CreERT2* (031820), *RCL-GCaMP6s* (028866), *R26-LSL-Gi-DREADD* (026219) and *R26-LSL-Gq-DREADD* (026220). All the experiments were performed on adult mice (2–6 months) of both sexes. A total of 4–8 mice were used for each experiment, and the specific numbers are included in figure legends. Mice of specific genotypes were randomly assigned to experimental and control groups. Experimental and control mice were subjected to exactly the same surgical and behavioral manipulations. Mice were housed in a 12-h light/12-h dark cycle (lights on at 7:00 a.m. and off at 7:00 p.m.) at constant room temperature and humidity with free access to food and water. All procedures were approved by the Animal Care and Use Committees of the University of California, Berkeley, and were conducted in accordance with federal regulations and guidelines on animal experimentation.

To generate mice with microglia-specific expression of G_i_–DREADD and GCaMP6s, we first bred *Tmem119-CreERT2* mice with *R26-LSL-Gi-DREADD* mice and then bred the *Tmem119-CreERT2*; *R26-LSL-Gi-DREADD* mice with *RCL-GCaMP6s* mice, resulting in *Tmem119-CreERT2*; *RCL-GCaMP6s*; *R26-LSL-Gi-DREADD* mice. A primer set targeting *GCaMP6s* was used to double-check the mouse genotype (5′-AGGACGACGGCAACTACAAG-3′ and 5′-CACCGTCGGCATCTACTTCA-3′). G_q_–DREADD mice were generated by breeding the *Tmem119-CreERT2* mice with *R26-LSL-Gq-DREADD* mice. To activate tamoxifen-inducible CreERT2, mice were gavaged at ~6 weeks of age with two doses of 250 mg kg^−1^ tamoxifen (T5648, Sigma) in sunflower seed oil (S5007, Sigma) with a separation of 48 h between doses.

### Virus preparation

AAV9-hsyn-GRAB_NE2m_ and AAV9-hsyn-GRAB_ADO1.0_ were obtained from WZ Biosciences. To construct the pAAV-Ef1α-DIO-p130PH-mCherry and pAAV-Ef1α-DIO-p130PH^R134L^-mCherry viral vectors, the transgene encoding p130PH or p130PH^R134L^ was amplified using PCR from the pEGFP-N1 plasmids containing these genes^35^ and inserted into the Ef1α-DIO-mCherry viral vector (Addgene, 47636). AAVTM6 viruses were produced at the Janelia Viral Tools facility, and the titer was >7 × 10^12^ genomic copies (gc) per ml. The titer of the NE and adenosine sensor viruses was estimated to be ≥1 × 10^13^ gc per ml.

### Surgery

For all the surgeries, adult mice were anesthetized with 1.5–2% isoflurane and placed on a stereotaxic frame. A heating pad was used to keep the body temperature stable throughout the procedure. Eye ointment was applied to keep the eyes from drying. After shaving hairs and asepsis with Betadine and medical alcohol, an incision was made to the skin to expose the skull.

For AAV injection, a craniotomy was made on top of the target region, and AAVs were injected into the target region using Nanoject II (Drummond Scientific) by a micropipette. Then, 2 μl of AAVTM6-Ef1α-DIO-p130PH-mCherry or AAVTM6-Ef1α-DIO-p130PH^R134L^-mCherry was injected into the lateral ventricle at the coordinates of anteroposterior (AP) +0.1 mm, mediolateral (ML) 0.9 mm and dorsoventral (DV) 2.5–2.8 mm. To activate p130PH or p130PH^R134L^ expression, mice were provided tamoxifen in chow for a 2-week period, starting at day 3 after virus injection (250 mg tamoxifen per kg of chow; Research Diets). Mice were gavaged with two additional doses of tamoxifen (250 mg kg^−1^ body weight) at days 7 and 9 after virus injection. For the NE and adenosine sensors, 0.25 μl of AAV9-hsyn-GRAB_NE2m_ or AAV9-hsyn-GRAB_ADO1.0_ was injected at each of two locations of the prefrontal cortex within the area 0.5–1.2 mm from the midline and anterior to the bregma, at a depth of 0.5 mm.

For implantation of the EEG and EMG recording electrodes, two miniature stainless-steel screws were inserted into the skull at AP −1 mm, ML 1.5 mm and AP −3 mm, ML 2.5 mm. Two EMG electrodes were inserted into the neck musculature. A reference screw was inserted into the skull on top of the right cerebellum. Insulated leads from the EEG and EMG electrodes were soldered to a pin header, which was secured to the skull using dental cement.

For i.c.v. and brain region-specific drug infusion, a bilateral cannula (Plastics One Technologies) was inserted into the lateral ventricle (AP +0.1 mm, ML 0.9 mm and DV 2.6 mm), the prefrontal cortex (AP +1.2 mm, ML 0.5 mm and DV 0.3 mm), the basal forebrain (AP +0.1 mm, ML 1.2 mm and DV 5.2 mm) and the dorsal striatum (AP +0.1 mm, ML 1.5 mm and DV 2.8 mm). The cannula was placed in the same surgery as EEM and EMG electrode implantation, and it was secured to the skull with dental cement.

For cranial window surgery, a dental drill (FST) with a diameter of 0.5 mm was used to drill through the skull for the craniotomy (3 × 3 mm^2^) over the prefrontal cortex. The craniotomy was centered at ~1 mm AP, 1 mm ML, and it covered part of the cingulate cortex and part of the secondary motor area. A 4 × 4­ mm^2^ glass coverslip (Warner Instruments) was cut using a diamond-point pen and attached to the 3 × 3 mm^2^ coverslip by UV glue (Norland Optical Adhesive, Norland). After being sterilized and dried, the combined coverslips were slowly lowered into the craniotomy. A cannula (Plastics One Technologies) was implanted carefully under the cranial window for local drug perfusion. Dental cement was applied around the window to cover the rim of the glass window. EEG and EMG electrodes were implanted as described above on the opposite side to the cranial window. A stainless-steel head-bar was then firmly attached to the skull, and all the remaining exposed skull surfaces were covered by dental cement. Mice were allowed to recover from anesthesia on a heating pad before being returned to their home cage. Meloxicam was provided as an analgesic for 24 h after surgery. In mice with AAV injections, windows were implanted over the injection site after a >2-week recovery period.

### Sleep recording

Behavioral experiments were mostly carried out in home cages placed in sound-attenuating boxes between 1:00 p.m. and 7:00 p.m. (with saline or CNO injection performed at ~1:00 p.m. and recording completed before 7:00 p.m.), except for those specifically tested in the early morning (Extended Data Fig. 2c–f,n; injection took place at ~8:00 a.m.) and the dark phase (Extended Data Fig. 2g–j,l,n; injection took place at ~8:00 p.m.). EEG and EMG electrodes were connected to flexible recording cables by a mini-connector. Recordings started after 20–30 min of habituation. The signals were recorded with a TDT RZ5 amplifier (OpenEx software suite), filtered (0–300 Hz) and digitized at 1,500 Hz. Spectral analysis was carried out using fast Fourier transform (FFT). For each 5-s epoch, the brain state was classified into NREM, REM or wake states on the basis of EEG and EMG data (wake: desynchronized EEG and high EMG activity; NREM: synchronized EEG with high-amplitude, low-frequency (0.5–4 Hz) activity and low EMG activity; REM: high power at theta frequencies (6–9 Hz) and low EMG activity). Consecutive epochs of the same state were combined into a single episode. The classification was performed semi-automatically using a custom-written graphical user interface (programmed in MATLAB, MathWorks). For the comparison of normalized EEG power spectra within each brain state, the EEG spectra were normalized to the total EEG power between 0 and 25 Hz.

### Chemogenetic and pharmacological manipulation

For chemogenetic manipulation, saline (0.9% sodium chloride) or CNO (C0832, Sigma; dissolved in saline) was injected i.p. into mice expressing G_i_–DREADD or G_q_–DREADD or mice without DREADD expression. The CNO dose was 1 mg kg^−1^ body weight for G_i_–DREADD or control experiments and 0.2 mg kg^−1^ or 1 mg kg^−1^ body weight for G_q_–DREADD experiments. Each recording session started immediately after injection and lasted for 5 h. Each mouse was recorded for 6–8 sessions, and CNO was given randomly in half of the sessions, while saline was given in the other half. Data were averaged across all sessions. For local chemogenetic manipulation, CNO (10 μM, 200 nl, dissolved in ACSF) or ACSF was infused into the target region through bilateral cannula, at 50 nl min^−1^ using a microinfusion pump. Each mouse was recorded for 6–8 sessions (CNO was given randomly in half of the sessions and ACSF was given in the other half) with an interval of at least 2 days. For i.c.v. drug infusion, PSB0739 (1 mM, 2 μl), 2MeSADP (300 μM, 2 μl), ICl (300 μM, 2 ul), a combination of phentolamine and propranolol (phentolamine, 500 μM; propranolol, 100 μM; 2 μl) or ACSF was infused into the lateral ventricle through cannula at 500 nl min^−1^.

### Two-photon imaging

Mice with a head-bar were first habituated to sleep under head-fixed conditions for two-photon imaging. To do this, the mice were kept head-fixed under the two-photon system for ~15 min, ~30 min and ~45 min for the first 3 days. The duration of head fixation increased by 20 to 30 min in each subsequent session, reaching a maximum of ~3 h. EEG and EMG signals were recorded during later sessions to monitor the state of the mouse until multiple wake–sleep cycles were observed.

During imaging sessions, the mouse was allowed about 10 min of habituation after being head-fixed before imaging started. For imaging with chemogenetic treatment, a 40-min baseline period was imaged before saline or CNO injection, and another 80 to 120 min was imaged after injection. For imaging with drug application, a 20-min baseline period was imaged before drug perfusion. Drug was perfused to the cortical surface through an infusion pump (Micro4, World Precision Instruments) at a rate of 0.5 μl min^−1^ (2-μl volume in total). The following drugs were used: ICl-118,551 (I127, Sigma), phentolamine mesylate (6431, Tocris), propranolol (P0884, Sigma), PSB0739 (3983, Tocris), 2MeSADP (1624/10, R&D) and 2-CADO (C5134, Sigma). All the drugs were constituted with ACSF (3525, Tocris).

Two-photon Ca^2+^, GRAB_NE2m_ or GRAB_ADO1.0_ imaging was performed using a custom two-photon microscope that was described previously^66^. EEG and EMG were recorded simultaneously with a TDT RZ5 amplifier as described above. The microscope (Movable Objective Microscope, Sutter Instrument) was controlled by ScanImage 3.8 software, and the objective was a ×20 water immersion lens (XLUMPlanFI, 0.95 NA, Olympus). A Mai-Tai Insight laser (Spectra-Physics) was tuned to 920 nm and ~35 mW output for microglia GCaMP6s imaging and <10 mW output for GRAB_NE2m_ and GRAB_ADO1.0_ imaging (as measured under the objective). Fluorescence emission was collected using a GaAsP PMT (H10770PA-40, Hamamatsu). Microglia Ca^2+^ was imaged at a frame rate of 0.84 Hz, 512 × 512 pixels and ×2 digital zoom. NE and adenosine signals were imaged at a frame rate of 1.68 Hz and pixel resolution of 256 × 256.

### Calcium imaging data analysis

Time series of Ca^2+^ activity images were motion-corrected with Inscopix Data Processing software. An average intensity image was then generated for ROI selection. Microglia with clear soma and processes were semi-automatically tracked using the Simple Neurite Tracer plugin in ImageJ. The morphology mask of the cell was obtained by applying the ‘Fill’ function within the plugin. Microglia soma and processes were then manually segmented into microdomains using the ‘freehand selections’ tool in ImageJ. Soma was identified on the basis of the size and the intensity. Processes were segmented if greater than 5 μm. The mean intensity values were generated with the multi-measure tool in ImageJ for the segmented ROIs. The mean fluorescence intensity and the standard deviation during the baseline period were used to obtain the *z*-score of the GCaMP6s signal. For the amplitude and frequency analysis, we adopted a previously described method^30^. Briefly, the baseline fluorescence of the ROI, *F*_0_, was determined as the lower 25th quartile of the fluorescence in a ±600-s sliding window and was used to calculate Δ*F*/*F*. A calcium transient was considered to have occurred at a threshold that was 3 s.d. above the mean value of the ∆*F*/*F* trace over three frames.

### GRAB_NE2m_ and GRAB_ADO1.0_ imaging data analysis

Time series of GRAB_NE2m_ or GRAB_ADO1.0_ images were motion-corrected using a custom MATLAB script from the ANTs open-source toolkit (https://picsl.upenn.edu/software/ants/). An average intensity image was then generated for ROI selection. Cell bodies were identified on the basis of the intensity. In regions without clear cell bodies, ROIs with diameters of 50–70 μm were selected. A total of 8–12 ROIs were manually selected across the image. The mean intensity of each ROI was generated over time. The mean fluorescence intensity and the standard deviation during the baseline period were used to obtain the *z*-score of GRAB_NE2m_ or GRAB_ADO1.0_ signals.

### Microglia–bouton distance analysis

For quantification of the distance between boutons and microglia, wild-type brain or brain with AAV2-EF1α-DIO-eYFP (250 nl, titer of ≥2 × 10^12^ gc per ml) injected into the locus coeruleus (AP −5.4 mm, ML 0.9 mm, DV 3.7 mm) of a *Dbh-Cre* mouse (036778-UCD, MMRRC) was perfused using PBS followed by 4% paraformaldehyde in PBS. Brains were post-fixed in 4% paraformaldehyde for 24 h. Further tissue processing, immunolabeling (with antibodies to TH, Iba1 and GFP), light-sheet imaging of the prefrontal cortex and image registration were performed by LifeCanvas Technologies with SmartSPIM at a 1-μm *z* step and an *x*–*y* pixel size of 0.41 μm. Regions with relatively even distribution of labeled axons were manually selected. Automatic denoising (with the same parameters) and manual adjustment of contrast and brightness were implemented as preprocessing steps to improve image quality through ImageJ software (NIH). Subsequently, DeepBouton^67^ was used to detect the boutons. An iterative weakly supervised segmentation network was also used to extract the microglia from the Iba1 images, which is an improved version of 3D Res-uNet^68^. Finally, the identified boutons were verified manually, and the minimal distance from an axon bouton to microglia was calculated. The 3D rendering of the example images in Fig. 6a and Extended Data Fig. 7a was generated with Imaris software (BITPLANE).

### Immunohistochemistry

For immunohistochemistry, mice were deeply anesthetized and transcardially perfused using PBS followed by 4% paraformaldehyde in PBS. Brains were post-fixed in 4% paraformaldehyde for 24–48 h and stored in 30% sucrose in PBS solution for 48 h for cryoprotection. Brains were embedded and mounted with Tissue-Tek OCT compound (Sakura finetek), and 30-μm sections were cut using a cryostat (Leica). For P2Y12 staining, brain slices were washed using PBS and subjected to heat-induced antigen retrieval with 0.1 M citric acid (pH 6.0) at 95 °C for 5 min. Slices were then washed with PBS, permeabilized using PBST (0.3% Triton X-100 in PBS) for 30 min and incubated with blocking solution (5% normal bovine serum in PBST) for 1 h, followed by rabbit anti-P2Y12 antibody (1:500; AS-55043A, AnaSpec) together with goat anti-Iba1 antibody (1;200; ab5076, Abcam) incubation overnight at 4 °C. The next day, after sufficient washing in PBS, sections were incubated in proper fluorescently conjugated secondary antibodies (1:500; Invitrogen) for 2 h at room temperature. For TMEM119 and Iba1 staining, the same procedure was used, except for the chicken anti-TMEM119 (1:500; 400006, Synaptic Systems) and rabbit anti-Iba1 (1:1,000; 019-19741, Fujifilm Wako) antibodies. Finally, sections were counterstained with DAPI (Sigma-Aldrich) and mounted on slides with VECTASHIELD Antifade Mounting Medium (Vector Laboratories, H-1000).

For HA staining, the Alexa Fluor 488 Tyramide SuperBoost Kit (B40922, Thermo Fisher Scientific) was used. Briefly, floating brain slices were treated with 3% hydrogen peroxide solution for 15 min to quench the endogenous peroxidase activity and then subjected to heat-induced antigen retrieval as described above. After washing with PBS, slices were incubated in blocking buffer (5% normal goat serum) for 1 h. Rabbit anti-HA antibodies (1:200; 3724, Cell Signaling Technology), together with chicken anti-Iba1 (1:1,500; 234009, Synaptic Systems), were applied overnight at 4 °C. Afterward, sections were washed three times with PBS for 10 min each. Alexa Fluor 647 anti-chicken IgG (1:500; A21449, Invitrogen), together with poly(HRP)-conjugated anti-rabbit secondary antibody, was applied to the slices for 2 h at room temperature. Slices were washed with PBS three times for 10 min each. Tyramide working solution was prepared according to the manufacturer’s instructions, and colors were developed with Alexa Fluor 488 Tyramide for 10 to 30 min. Reaction stop reagent was applied, and slices were counterstained with DAPI and mounted with antifade mounting medium.

For p130PH–mCherry staining, brain slices were washed using PBS, permeabilized using PBST for 30 min and incubated with blocking solution (5% normal goat serum in PBST) for 1 h. Rat anti-mCherry antibody (1:300; M11217, Life Technologies), together with chicken anti-Iba1 and rabbit anti-P2Y12 antibodies, was applied overnight at 4 °C. The next day, after sufficient washing in PBS, sections were incubated with Biotin-SP-conjugated anti-rat (1:500; Jackson ImmunoResearch), Alexa Fluor 647 anti-chicken (1:500) and Alexa Fluor 488 anti-rabbit (1:500; A-21206, Invitrogen) secondary antibodies for 2 h at room temperature. Sections were sufficiently washed again, and Alexa Fluor 594-conjugated streptavidin (1:1,000; Jackson ImmunoResearch) was applied to the slices for 1.5 h at room temperature. Afterward, slices were counterstained with DAPI and mounted with antifade mounting medium.

For the quantification of marker colocalization, fluorescence images were taken with a confocal microscope (Zen 2010 software, LSM 710 AxioObserver Inverted 34-Channel Confocal, Zeiss, ×20, for Figs.1 and 3 and Extended Data Figs. 1 and 4). For general histology (Extended Data Figs. 6c and 7c,e,j,o), a fluorescence microscope (Keyence BZX-710, ×20) was used. Manual cell counting was performed with a custom-written graphical user interface programmed in MATLAB software in a region of ~1,000 μm × 500 μm to 1,000 μm × 1,000 μm in each brain area. Cells expressing both markers (for example, Iba1 and P2Y12 in Fig. 1j, HA and Iba1 in Extended Data Fig. 1b, TMEM119 and Iba1 in Extended Data Fig. 1d and mCherry and Iba1 in Extended Data Fig. 4b) were defined as ‘Overlap’.

### Statistics and reproducibility

Statistical analysis was performed using GraphPad Prism, and significance was determined at *P* < 0.05. All statistical tests were two-sided. The selection of statistical tests was based on previously reported studies. No statistical method was used to predetermine sample size, but our sample sizes are similar to those reported in previous publications^15,22,42,43^. A normality test was performed on each dataset using the Shapiro–Wilk test or D’Agostino and Pearson test. For comparison of two group means, parametric tests (paired *t-*test or unpaired *t-*test) were used if the dataset was normally distributed; otherwise, nonparametric tests (Wilcoxon signed rank test or Mann–Whitney *U* test) were used. One-way ANOVA was used for comparison across more than two groups. Two-way ANOVA with Bonferroni correction was used for comparisons of brain state between different conditions (saline versus CNO) in chemogenetic experiments. All the representative images and recording traces provided in the figures reflect a minimum of three biological replicates with similar results. Mice of specific genotypes were randomly assigned to experimental and control groups, and each mouse of a specific genotype was subjected to both control and experimental treatment. Data collection and analysis were not performed blind to the conditions of the experiments; however, data analysis was performed with the same parameters and as automated as possible. Only a few sessions in two-photon imaging, in which drug application caused large motion (based on landmarks in the imaging window), were excluded. All other data were included.

### Reporting summary

Further information on research design is available in the Nature Portfolio Reporting Summary linked to this article.

## Online content

Any methods, additional references, Nature Portfolio reporting summaries, source data, extended data, supplementary information, acknowledgements, peer review information; details of author contributions and competing interests; and statements of data and code availability are available at 10.1038/s41593-023-01548-5.

### Supplementary information

Reporting Summary

Supplementary Video 1 Two-photon imaging of Ca^2+^ activity of a representative microglia before and after CNO-induced G_i_–DREADD activation in *Tmem119-CreERT2;* *RCL-GCaMP6s*; *R26-LSL-Gi-DREADD* mice, related to data shown in Fig. 2c–f and Extended Data Fig. 3.

Supplementary Code 1 Supplementary Code 1.

Supplementary Code 2 Supplementary Code 2.

Supplementary Code 3 Supplementary Code 3.

Supplementary Code 4 Supplementary Code 4.

Supplementary Code 5 Supplementary Code 5.

Supplementary Code 6 Supplementary Code 6.

Supplementary Code 7 Supplementary Code 7.

Supplementary Code 8 Supplementary Code 8.

### Source data

Source Data Fig. 1 Statistical source data.

Source Data Fig. 2 Statistical source data.

Source Data Fig. 3 Statistical source data.

Source Data Fig. 4 Statistical source data.

Source Data Fig. 5 Statistical source data.

Source Data Fig. 6 Statistical source data.

Source Data Extended Data Fig. 1 Statistical source data.

Source Data Extended Data Fig. 2 Statistical source data.

Source Data Extended Data Fig. 3 Statistical source data.

Source Data Extended Data Fig. 4 Statistical source data.

Source Data Extended Data Fig. 5 Statistical source data.

Source Data Extended Data Fig. 6 Statistical source data.

Source Data Extended Data Fig. 7 Statistical source data.

## Data Availability

Source data are provided with this paper.

## Code availability

MATLAB scripts used to analyze brain state probability, episode duration and number, and manual cell counting are available as supplementary information.
