# Supplementary material for: Microglia regulate sleep through calcium-dependent modulation of norepinephrine transmission
Source: Nat Neurosci. 2024 Jan 18;27(2):249–58. doi: 10.1038/s41593-023-01548-5 (PMC10849959; doi:10.1038/s41593-023-01548-5)
Supplement: Supplementary file 1 — Reporting Summary [file 41593_2023_1548_MOESM1_ESM.pdf]

nature portfolio

Corresponding author(s): Yang Dan

Last updated by author(s): Sep 7, 2023

Reporting Summary

Nature Portfolio wishes to improve the reproducibility of the work that we publish. This form provides structure for consistency and transparency in reporting. For further information on Nature Portfolio policies, see our Editorial Policies and the Editorial Policy Checklist.

Statistics

For all statistical analyses, confirm that the following items are present in the figure legend, table legend, main text, or Methods section.

n/a	Confirmed	
☐	☒	The exact sample size (*n*) for each experimental group/condition, given as a discrete number and unit of measurement
☐	☒	A statement on whether measurements were taken from distinct samples or whether the same sample was measured repeatedly
☐	☒	The statistical test(s) used AND whether they are one- or two-sided<br>*Only common tests should be described solely by name; describe more complex techniques in the Methods section.*
☐	☒	A description of all covariates tested
☐	☒	A description of any assumptions or corrections, such as tests of normality and adjustment for multiple comparisons
☐	☒	A full description of the statistical parameters including central tendency (e.g. means) or other basic estimates (e.g. regression coefficient) AND variation (e.g. standard deviation) or associated estimates of uncertainty (e.g. confidence intervals)
☐	☒	For null hypothesis testing, the test statistic (e.g. *F*, *t*, *r*) with confidence intervals, effect sizes, degrees of freedom and *P* value noted<br>*Give P values as exact values whenever suitable.*
☒	☐	For Bayesian analysis, information on the choice of priors and Markov chain Monte Carlo settings
☒	☐	For hierarchical and complex designs, identification of the appropriate level for tests and full reporting of outcomes
☐	☒	Estimates of effect sizes (e.g. Cohen's *d*, Pearson's *r*), indicating how they were calculated

Our web collection on statistics for biologists contains articles on many of the points above.

Software and code

Policy information about availability of computer code

Data collection	Confocal images were taken with Zen 2010 software. Sleep recordings were done with OpenEx software suite (v2.20) (Tucker-Davis Technologies). Two-photon imaging data were collected with ScanImage 3.8, an open source software. Light sheet imaging was done by LifeCanvas Technologies with SmartSPIM light sheet microscope using SmartSPIM's acquisition software (please check LifeCanvas Technologies website for more details).
Data analysis	Softwares and scripts used are described in the Methods section and listed below as well.<br>Data analysis: Confocal images were processed with ImageJ (v 2.3.0), and manual cell counting was done with a custom-written graphical user interface programmed in MATLAB software (MATLAB, R2019b). Brain state classification was done semi-automatically using a custom-written graphical user interface programmed in MATLAB (as reported in our previous publications). Two-photon imaging data were processed with Inscopix Data Processing software (v 1.3.1), Advanced Normalization Tools (ANTs) toolkits, ImageJ (v 2.3.0), and MATLAB. Statistics were done with GraphPad Prism 9.2.0. Axon boutons were automatically detected by DeepBouton.<br>Data representation: Figures were prepared with GraphPad Prism 9.2.0, Adobe InDesign 2021, and Adobe Illustrator 2021. The movie was prepared with Adobe Premiere Pro 2022. The 3D rendering of example images were generated with Imaris software (BITPLANE).

For manuscripts utilizing custom algorithms or software that are central to the research but not yet described in published literature, software must be made available to editors and reviewers. We strongly encourage code deposition in a community repository (e.g. GitHub). See the Nature Portfolio guidelines for submitting code & software for further information.

Data

Policy information about availability of data

All manuscripts must include a data availability statement. This statement should provide the following information, where applicable:

- Accession codes, unique identifiers, or web links for publicly available datasets
- A description of any restrictions on data availability
- For clinical datasets or third party data, please ensure that the statement adheres to our policy

The dataset used for figures are available as supplementary information.

Research involving human participants, their data, or biological material

Policy information about studies with human participants or human data. See also policy information about sex, gender (identity/presentation), and sexual orientation and race, ethnicity and racism.

Reporting on sex and gender	NO human research participants in the study.
Reporting on race, ethnicity, or other socially relevant groupings	NO human research participants in the study.
Population characteristics	NO human research participants in the study.
Recruitment	NO human research participants in the study.
Ethics oversight	NO human research participants in the study.

Note that full information on the approval of the study protocol must also be provided in the manuscript.

Field-specific reporting

Please select the one below that is the best fit for your research. If you are not sure, read the appropriate sections before making your selection.

☒ Life sciences ☐ Behavioural & social sciences ☐ Ecological, evolutionary & environmental sciences

For a reference copy of the document with all sections, see nature.com/documents/nr-reporting-summary-flat.pdf

Life sciences study design

All studies must disclose on these points even when the disclosure is negative.

Sample size	No statistical methods were used to predetermine the sample sizes. Sample sizes were determined based on previous studies (e.g., Liu et al., 'A common hub for sleep and motor control in the substantia nigra', Science, 2020) and lab experience.
Data exclusions	Only a few sessions with drug application causing large motion (based on landmarks in the imaging window) were excluded. Otherwise, no data were excluded.
Replication	Each experiment was done with multiple mice and each mouse was tested for multiple sessions. All attempts at replication of experiments were successful, as evident by the individual data points reported throughout the figures.
Randomization	Mice of specific genotype were randomly assigned to experimental and control groups. Each mouse of a specific genotype was subjected to both control and experimental treatment.
Blinding	Investigators were not blinded for group allocations since each mouse of a specific genotype was subjected to both control and experimental treatment. For the NE imaging data, the behavioral data and the imaging data were processed independently by two researchers.

Reporting for specific materials, systems and methods

We require information from authors about some types of materials, experimental systems and methods used in many studies. Here, indicate whether each material, system or method listed is relevant to your study. If you are not sure if a list item applies to your research, read the appropriate section before selecting a response.

Materials & experimental systems

n/a	Involved in the study
☐	☒ Antibodies
☒	☐ Eukaryotic cell lines
☒	☐ Palaeontology and archaeology
☐	☒ Animals and other organisms
☒	☐ Clinical data
☒	☐ Dual use research of concern
☒	☐ Plants

Methods

n/a	Involved in the study
☒	☐ ChIP-seq
☒	☐ Flow cytometry
☒	☐ MRI-based neuroimaging

Antibodies

Antibodies used

Information of all antibodies used were given in the Methods section and listed below as well.
Primary antibodies:
rabbit anti-HA antibodies (1:200 dilution) (#3724, Cell Signaling Technology);
chicken anti-Iba1 antibodies (1:1500 dilution) (#234009, Synaptic Systems)
goat anti-Iba1 antibodies (1:200 dilution) (ab5076, Abcam);
rabbit anti-P2Y12 antibodies (1:500 dilution) (AS-55043A, AnaSpec);
Rat anti-mCherry antibodies (1:300 dilution) (M11217, Life Technologies)
chicken anti-TMEM119 antibodies (1:500 dilution)((#400006, Synaptic Systems)
rabbit anti-Iba1 antibodies (1:1000 dilution) ((#019-19741, Fujifilm Wako)

Secondary antibodies:
anti-goat IgG-Alexa Fluor 594 (1:500 dilution)(A-11058, Invitrogen);
anti-rabbit IgG-Alexa Fluor 488 (1:500 dilution)(A-21206, Invitrogen);
Alexa Fluor 488 Tyramide SuperBoost Kit (B40943, Invitrogen);
anti-chicken Alexa Fluor 647 (1:500 dilution)(A21449, Invitrogen);
Biotin-SP-conjugated anti-rat (1:500 dilution)(712-065-153, Jackson ImmunoResearch);
Alexa Fluor 594-conjugated streptavidin (1:1000 dilution)(016-580-084, Jackson ImmunoResearch)
anti-rabbit IgG-Alexa Fluor 546 (1:500 dilution) (A10040, Invitrogen)
anti-chiken IgG-Alexa Fluor 488 (1:500 dilution) (703-545-155, Jackson ImmunoResearch)
Alexa Fluor 488 Tyramide SuperBoost Kit with Poly-HRP-conjugated anti-rabbit secondary antibody (B40922, Thermo Fisher Scientific)

Validation

All antibodies were validated for immunostaining by the manufactures on their websites or by previous studies. For example:

rabbit anti-HA antibodies ((#3724, Cell Signaling Technology) (validated with HA-transfected and untransfected cells on the manufacture's website)
Chicken anti-Iba1 antibodies (#234009, Synaptic Systems): reacts with: rat, mouse, , human, ape. Other species not tested yet.
goat anti-Iba1 antibodies (ab5076, Abcam): reacts with: Rat, Human; predicted to work with: Pig, Macaque monkey
rabbit anti-P2Y12 antibodies (AS-55043A, AnaSpec): reactivity with mouse
Rat anti-mCherry antibodies (M11217, Life Technologies): validated with mcherry transfected cells on the manufacture's website
chicken anti-TMEM119 antibodies ((#400006, Synaptic Systems): reacts with mouse. Other species not tested yet.
rabbit anti-Iba1 antibodies ((#019-19741, Fujifilm Wako): react with Human, Mouse, Rat

Animals and other research organisms

Policy information about studies involving animals; ARRIVE guidelines recommended for reporting animal research, and Sex and Gender in Research

Laboratory animals

All mouse lines were purchased from Jackson Laboratory or Mutant Mouse Resource and Research Center (MMRRC) and maintained on a C57BL/6J background. The following lines were used in this study (Jackson stock number or MMRRC number in parenthesis): Tmem119-2A-CreERT2 (031820), RCL-GCaMP6s (028866), R26-LSL-Gi-DREADD (026219), R26-LSL-Gq-DREADD (026220)., Dbh-Cre (036778-UCD) . 2-6 month-old mice of both sexes were used in this study.

Wild animals

This study did not involve wild animals.

Reporting on sex

Both male and female mice were used in this study.

Field-collected samples

This study did not involve samples collected from the field.

Ethics oversight

All procedures were approved by Animal Care and Use Committees of the University of California, Berkeley and were done in accordance with federal regulations and guidelines on animal experimentation.

Note that full information on the approval of the study protocol must also be provided in the manuscript.

April 2023
